# Regulation of a Truncated Form of Tropomyosin-Related Kinase B (TrkB) by Hsa-miR-185* in Frontal Cortex of Suicide Completers

**DOI:** 10.1371/journal.pone.0039301

**Published:** 2012-06-25

**Authors:** Gilles Maussion, Jennie Yang, Volodymyr Yerko, Philip Barker, Naguib Mechawar, Carl Ernst, Gustavo Turecki

**Affiliations:** 1 McGill Group for Suicide Studies, Douglas Hospital Research Institute, McGill University, Montreal, Quebec, Canada; 2 Montreal Neurological Institute, McGill University, Montreal, Quebec, Canada; Deutsches Krebsforschungszentrum, Germany

## Abstract

**Background:**

TrkB-T1 is a BDNF receptor lacking a tyrosine kinase domain that is highly expressed in astrocytes and regulates BDNF-evoked calcium transients. Previous studies indicate that downregulation of TrkB-T1 in frontal cortex may be involved in neurobiological processes underlying suicide.

**Methods:**

In a microarray screening study (N = 8), we interrogated all known microRNA in the frontal cortex of suicide completers with low expression of TrkB-T1 and normal controls. These findings were validated and followed up in a larger sample of cases and controls (N = 55). Functional analyses included microRNA silencing, microRNA overexpression and luciferase assays to investigate specificity and to validate interactions between differentially expressed microRNA and TrkB-T1.

**Results:**

MicroRNAs Hsa-miR-185* and Hsa-miR-491-3p were upregulated in suicide completers with low expression of TrkB.T1 (P_nominal_: 9.10^−5^ and 1.8.10^−4^ respectively; FDR-corrected p = 0.031). Bioinformatic analyses revealed five putative binding sites for the DiGeorge syndrome linked microRNA Hsa-miR-185*in the 3′UTR of TrkB-T1, but none for Hsa-miR-491-3P. The increase of Hsa-miR-185* in frontal cortex of suicide completers was validated then confirmed in a larger, randomly selected group of suicide completers, where an inverse correlation between Hsa-miR-185* and TrkB-T1 expression was observed (R = −0.439; p = 0.001). Silencing and overexpression studies performed in human cell lines confirmed the inverse relationship between hsa-mir-185* and trkB-T1 expression. Luciferase assays demonstrated that Hsa-miR-185* binds to sequences in the 3′UTR of TrkB-T1.

**Conclusion:**

These results suggest that an increase of Hsa-miR-185* expression levels regulates, at least in part, the TrkB-T1 decrease observed in the frontal cortex of suicide completers and further implicate the 22q11 region in psychopathology.

## Introduction

Suicide is a leading cause of death in many regions of the world, with prevalence estimates ranging from 10–20 deaths per 100,000 people [1]. Suicide rates are highest in young men, but affect all people regardless of age, sex, or race. Clinical studies have consistently determined that suicide is most often associated with an underlying mood disorder, most commonly depression-related psychopathology [2], [3], [4], [5], [6], [7], [8], yet molecular studies have so far been unable to link any biological marker to suicide cases across independent samples.

Neurotrophic tyrosine kinase receptor type 2 (NTRK2) and ligand brain-derived neurotrophic factor (BDNF) are the subject of intense investigation in psychiatry and have been associated with anxiety, suicide, and depression, amongst other disorders [9], [10]. Currently, three major isoforms of the *NTRK2* gene located at 9q21.3 have been identified and each varies in length at the 3′ end. While each of these three transcripts, referred to as *TrkB-T1*, *TrkB-T2* and *TrkB-FL*, code for receptors capable of ligand binding, only the full-length 24 exon isoform (FL) has a domain with tyrosine kinase activity. *TrkB-T1* and *TrkB-T2* have stop codons at exons 16 and 19, respectively. Expression patterns in brain differ across NTRK2 isoforms, in particular, with the exclusive expression of TrkB-T1 in astrocytes [11].

Recently, we reported that *TrkB-T1* is markedly downregulated in the frontal cortex of a sub-group of individuals who died by suicide [12], [13].These results are consistent with recent findings suggesting that astrocytes play an important role in cellular communication [14] and with growing evidence pointing to astrocytic dysfunction in the pathophysiology of mood disorders [15], [16], [17] and other psychiatric disorders [18]. To understand the downregulation of TrkB-T1 in frontal cortex of suicide completers, we investigated genetic and epigenetic variation at the *TrkB* promoter. While promoter sequencing revealed no variants that could explain the finding (unpublished observations), we found that epigenetic marks could, in part, explain the decrease in *TrkB-T1* expression. We reported that two CpG islands in the *NTRK2* promoter were hypermethylated in the suicide brain and that these findings significantly correlated with *TrkB-T1* expression levels [12]. We have also shown that histone 3 lysine 27 methylation contributed to the decreased *TrkB-T1* expression levels in the suicide brain [19]. Although these epigenetic modifications are interesting mechanisms to explain the reported *TrkB-T1* downregulation in suicide, they do not fully account for the total variance in TrkB-T1 expression changes. Therefore, additional regulatory processes may be underlying the *TrkB-T1* downregulation observed in suicide.

MicroRNAs, best described as post-transcriptional regulators, are excellent candidates for the regulation of specific gene transcripts, and as such, could contribute to the down-regulation of *TrkB-T1* observed in frontal cortex of suicide completers. MicroRNAs are non-coding RNAs of an average length of 21 bp that function to inhibit the translation of messenger RNA and induce its destruction by binding to its 3′UTR sequence [20]. Prior to its functional form, microRNAs undergo a process of maturation. A primary microRNA is synthesized by the RNA polymerase ll and once cleaved by the Drosha/DGCR8 complex, the primary microRNA produces a pre-microRNA in the form of a hairpin loop [21]. The TRBP/Dicer complex then cleaves this hairpin resulting in a RNA/RNA duplex [22].

In this study, we explored the potential role of micro-RNAs in the regulation of *TrkB-T1* expression in the suicide brain. Through a series of complementary experiments beginning with a global microRNA microarray screening, we show that the expression of Hsa-miR-185* is significantly increased in suicide cases with low TrkB-T1 cortical expression. After replicating the association between Hsa-miR-185* and *TrkB-T1* brain expression in an independent and significantly larger sample, we conducted a series of functional studies showing that Hsa-miR-185* binds to *TrkB-T1* 3′UTR sequences and specifically affects its expression.

## Materials and Methods

### Subjects

This study was approved by the Douglas Institute Research Ethics Board, and all participating families signed consent forms. Brain tissue was obtained from the Quebec Suicide Brain Bank (QSBB:www.douglasrecherche.qc.ca/suicide). The initial microRNA screening was conducted in a sample selected according to *TrkB-T1* levels. This sample consisted of eight subjects, including four suicides with low levels of TrkB-T1 expression, and four normal controls. The replication sample used comprised a total of 55 individuals, including 38 suicides and 17 controls. All subjects were male and of French Canadian origin, a homogeneous population with a well-defined founder effect. All subjects died suddenly and could not have undergone any resuscitation procedures or other type of medical intervention, and thus did not experience prolonged agonal states. The majority of suicide completers had a history of major depressive disorder (N = 23, 60.5%), followed by schizophrenia (N = 4, 10.5%) and bipolar disorder (N = 1, 2.6%). Six (15.8%) did not have evidence of psychopathology and all controls were psychiatrically normal.

### Diagnostic Procedures

Diagnoses were obtained by means of psychological autopsies performed by trained clinicians using the SCID-I [23] with the informant best-acquainted with the deceased, as described elsewhere [24]. Briefly, this is a structured diagnostic procedure to elicit diagnostic information by means of proxy-based interviews complemented with medical and coroner records, followed by a consensus diagnosis reached by a panel of clinicians [25], [26]. We elicited DSM-IV [27] psychiatric diagnoses.

### Tissue Processing

With the aid of a human brain atlas [28], expert QSBB staff dissected samples from Brodmann area 10 (BA10), one of the frontal cortex regions previously shown to display *TrkB-T1* expression differences between suicides and controls [12]. RNA was extracted using miRNeasy kit (Qiagen Inc., Mississauga, Canada) following procedures suggested by the manufacturer. Total RNA was used for the microarray experiment. For validation and all other microRNA quantifications performed by real time PCR, a microRNA enriched fraction obtained with RNeasy MinElute Kit (Qiagen Inc.) was used. Sample mean RNA Integrity Number(RIN) values was 6,9±0,1.

### MicroRNA Arrays

The Exiqon miCURY™ v10.0 LNA was used for microRNA arrays, as it contains all 757 microRNAs probes characterized at the time this study was conducted (miRBase_v.11). Each sample was labelled with Hy3 and hybridized with a reference sample labelled with Hy5 using miRCURY™ LNA Array Power labeling kit (miRCURY LNA microRNA Array, v.10.0). Reference sample was made as a pool of equal quantities of 8 total RNA samples used in this experiment. Hybridization, scanning and normalization were performed according to the manufacturer’s standard protocol (Exiqon, MA,USA). A background correction was conducted according to Normexp with offset value 10 [29]. Data were transformed (log2) and normalized with the LOESS method [30].

### Reverse Transcription

The microRNA Reverse transcription kit (Applied Biosystems, CA,USA) and specific primers (#001093: RNU6B,# 002104: Hsa-miR-185*, #002340: Hsa-miR-423-5p and #002281: Hsa-miR-193a-5p; Applied Biosystems) were used to generate cDNA from microRNA enriched fraction previously extracted. Reverse transcriptions were done on total RNA fraction in order to obtain cDNA in 40 µl volume containing 1 µg of total RNA; 0,5 µg oligoDT(16) primers, 0.5 mM dNTPs, 0,01 M DTT and 400 U M-MLV RT (Carlsbad, CA).

### Sequences Analysis

The RNA22 http://cbcsrv.watson.ibm.com/rna22_targets.html database was used to characterize differential expressed microRNAs that could bind *TrkB-T1* 3′UTR sequence [31].

### Real Time PCR

Relative quantification of MicroRNAs: The reactions were performed in a total volume of 20 µl, on 384 well plates using an Applied Biosystems 7900 HT. We used mix and specific primers commercialized by Applied Biosystems. We used a reference pool of cDNA to generate a standard curve for RNU6B quantification. RNU6B, which is the U6B small nuclear RNA, is a commonly used internal control (reference to adjust for sample fluctuations ) used for quantification of microRNA. In order to quantify Hsa-miR-185* microRNA, a cDNA was generated from a synthetic RNA made by IDT. Serial dilutions provided amounts ranging between 0.78125 ng and 25 ng for Hsa-miR-185* and between 0.012207 ng and 50 ng for RNU6B. For each well, PCR mix included 10 µl of 2X NoAmperaseUNG mastermix (Applied Biosystems), 1 µl of primers/probe mix, 2 µl of cDNA, H_2_0 qsp 20 µl.

mRNA quantification: These reactions were conducted as described above, but in a total volume of 12 µl. Serials dilution provided amounts ranging between 0.003052 ng and 50 ng. Each well included 6 µl of 2X gene expression master mix, 0.6 µl of 20X primer mix, 3.4 µl of RNase free water and 2 µl of cDNA. βactin was used as internal control for normalization.

### Cloning

Fragments of *TrkB-T1* 3′UTR sequence containing putative binding sites for Hsa-miR-185* were obtained by PCR (Supporting Table S1). Those fragments were cloned in a TOPO TA Cloning vector using the procedure described by the manufacturer. A second PCR using primers with restriction sites was performed. Both fragments and PMIR-report vector (AMBION Austin, TX,) were digested and purified. The ligation was done at 15°C for 3 hours using T4 ligase (New England Biolabs). The ligation product was transformed in TOP10 competent cells (InVitrogen).

### Cell Lines

Two cell lines were used for functional analyses.Human embryonic kidney (HEK293) and neuroblastoma cell line (CRL2137). HEK293 cells were a gift from Dr Nicolas Cermakian [32]. CRL2137 were provided by ATCC (Manassas, VA; USA). Both of them were cultured in Dulbecco’s Modified Eagle’s Medium (DMEM) with 10% fetal bovine serum in 100 mm dishes.

### Transfection

CRL2137 cells were seeded in 6 well plates and transfected with an antagomir. RNA extraction procedures began eight hours post-transfection. Antagomirs MIN0004611 and #1027271 (Qiagen), respectively for Hsa-miR-185* and a manufacturer supplied negative control were transfected individually at 25 nM (final concentration), in CRL2137 cells using Hiperfect reagent (Qiagen) as recommended by the manufacturer. HEK293 cells were transfected with Mimics (Double-stranded oligonucleotide RNAs that mimic mature endogenous miRNAs) 24hours after seeding in 6 well plates - MSY0004611 and #1027280 (Qiagen), respectively for Hsa-miR-185* and negative control, at 10 nM (final concentration) and RNA extraction procedures began 24hours post-transfection.

For luciferase assays cells were transfected 24hours after seeding with (i) 60 ng of construct with or without *TrkB-T1* vector, (ii) 8 ng of pRL-Null vector, used to normalize the efficiency of transfection, (iii) and a mimic or a negative control at a final concentration of 10 nM. Proteins were extracted 24 hours after transfection and the luciferase activity was quantified using a luminometer berthold and dual luciferase assay kit (Promega).

### Statistical Analyses

Post-normalized data from the microarray study were treated with Partek Genomic Suite Software. T tests were performed on each probe in between group, giving nominal Pvalues. Then corrections for multiple testing were made using False Discovery Rate (FDR 0.05) [33]. Data was log transformed when appropriate, as commonly done in expression studies. Student t tests ANOVAs and ANCOVA were conducted as appropriate. For student t tests involving groups with variances significantly different, a Welch correction that potentially affects degrees of freedom, was applied. Similarly, correlation studies were conducted according to the distribution of the data. RT-PCR data were acquired using the SDS software version 2.2.2. All analyses were performed using SPSS version 18 software.

## Results

### microRNA Microarray Screening Study

**Figure 1:**
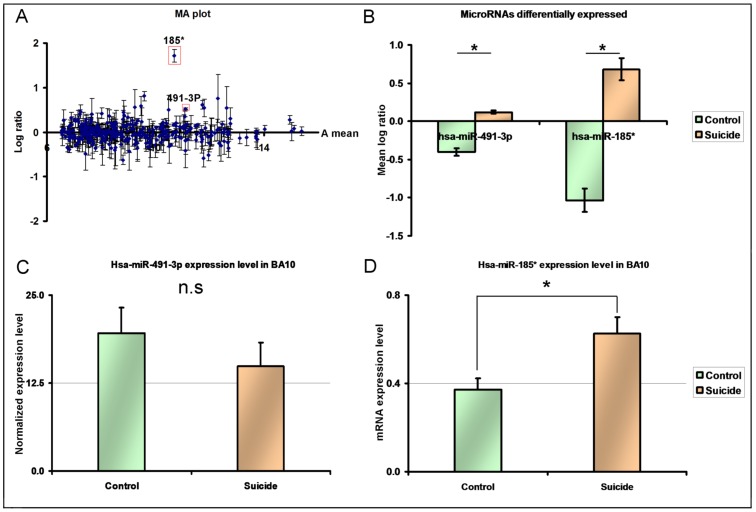
Microarray investigation and real-time PCR validation of microRNA differentially expressed in BA10 tissue from a screening sample composed of suicide completers selected according to extreme low *TrkB-T1* levels and controls with normal *TrkB-T1* expression values (N = 8). A: MA plot of the average probeset differential expression between groups. Hsa-miR-185* and Hsa-miR-491-3P are indicated by a red square. B: MicroRNAs differentially expressed after correction for multiple testing (FDR : 0.05) C: Quantification of Hsa-miR-491-3P in the microarray samples by real time PCR. D: Validation of Hsa-miR-185* in the microarray samples by real time PCR. Comparison was done by t test. *: p<0.05. n.s: not significant. Errors bars indicate standard errors of the mean.

To understand how microRNA’s affect expression of *TrkB-T1* in the frontal cortex of suicide completers, we performed an exploratory global microarray expression screen for all available microRNA’s in the brains of suicide completers with low levels of *TrkB-T1* and controls matched for age, gender, Post-Mortem Interval (PMI), and Potential of Hydrogen (pH) (N = 8; Supporting Table S2) [12]. Of the 757 probes on the array, 350 were expressed in BA10 (Supporting Table S3) (Figure 1A). Following FDR statistical correction for multiple tests, Hsa-miR-491-3P and Hsa-miR 185* were found to be significantly differentially expressed and upregulated: (P_nominal_:9.10^−5^ and 1.8.10^−4^ respectively; FDR-corrected p = 0.031 for both) Figure 1B.

Given that suicide completers were selected on the basis of low *TrkB-T1* expression, it follows that Hsa-miR-491-3P and/or Hsa-miR 185* could affect the expression of *TrkB-T1*. To determine whether these microRNAs were good candidates for the regulation of TrkB-T1 expression, we sought putative binding sites in the 3′UTR sequence of *TrkB-T1*. *In silico* investigation identified five putative binding sites for Hsa-miR-185* in the 3′UTR sequence of *TrkB-T1*, whereas no sites were identified for Hsa-miR-491-3P (Figure S1).

In order to determine the most accurate endogenous control for microRNA quantification, we measured the expression levels of RNU6B, RNA5S and Hsa-miR-26b as well as Hsa-miR-185* expression level on a subset of patients. We then compared the coefficient of variation (CV) defined as the ratio of standard deviation out of mean for the three sets of normalized data. The lowest coefficient of variation was found for Hsa-miR-185* expression levels normalized with RNU6B (40.9% compared to 43.1% for Hsa-miR-26b and 112.6% for RNA5S). We also compared the expression level of RNU6B in BA10 from controls and suicide completers and found no difference between groups (t = 1.3 df = 53 p = 0.199). We consequently decided to perform all microRNA quantifications with RNU6B as endogenous control.

RT-PCR investigation of Hsa-miR-185* expression in frontal cortex samples of cases and controls included in the initial array study showed a positive correlation (R^2^ = 0.6638 p = 0.014) with array results and validated a significant) increase of this microRNA among suicides (Figure 1D, t = 2.861, df = 6, p = 0.028), while the differential expression of Hsa-miR-491-3P was not validated by RT-PCR (Figure 1C, t = 0.9476, df = 6, p = 0.379). Hsa-miR-185* is thus a good candidate microRNA to regulate *TrkB-T1* expression, and therefore, we decided to further investigate its potential role in the neurobiology of suicide.

### Analysis of *TrkB-T1* Expression in a Larger Patient Sample

**Figure 2:**
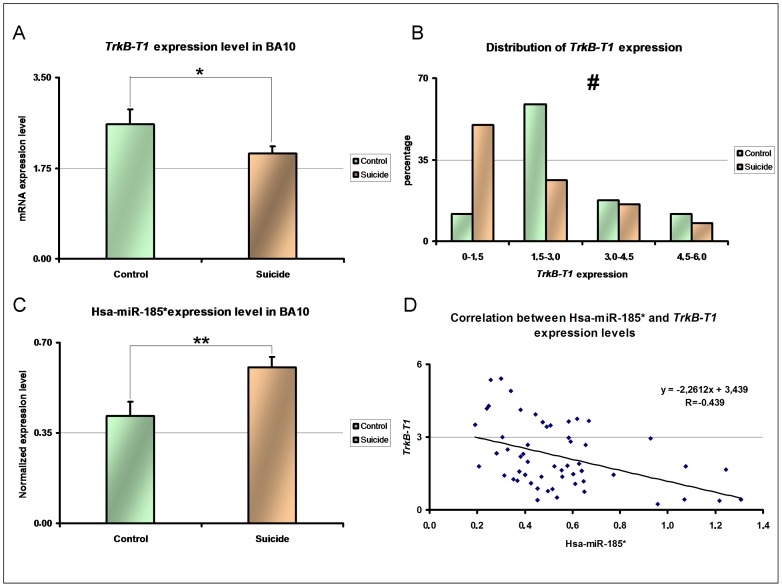
Investigation of *TrkB-T1* and Hsa-miR-185* expression levels in BA10 tissue from an independent replication sample composed of 17 controls and 38 suicide completers. A: Decrease of *TrkB-T1* expression level in BA10 tissue from suicides completers (N = 38) compared to controls (N = 17) observed by t test. B: Ranking of normalized *TrkB-T1* expression levels in controls (N = 17) and suicide completers (n = 38) tested by Chi Square test. # p<0.05 C: Increase of Hsa-miR-185* expression levels in BA10 from suicide completers (N = 38) and controls (N = 17) observed by t test. D: Significant correlation between Hsa-miR-185* and *TrkB-T1* expression levels in BA10 from the 55 patients investigated observed by Pearson correlation. *: p<0.05 **:p<0.01 Error bar indicate standard errors of the mean.

With the aim of investigating the generalizability of the array experiment results, we subsequently examined by RT-PCR the *TrkB-T1* expression levels in frontal cortex from an independent sample of 55 individuals. This sample included 38 male suicide completers and 17 male controls (Supporting Table S4). These subjects were selected on the basis of tissue quality, but *TrkB-T1* expression levels were unknown. We observed a significant reduction in *TrkB-T1* levels among suicides (t = −2.665, df = 53, p = 0.011) Figure 2A, which was not explained by possible confounders such as age, pH or PMI and did not differ according to violent and non-violent suicide method (p>0.05 for all factors).

In addition, our previous observations suggest an extreme downregulation of TrkB-T1 in a subgroup of suicide completers [12]. As expected the distribution of normalized expression levels of the *TrkB-T1* transcript obtained by quantitative PCR, grouped by class (Figure 2B), was significantly different in the suicide group compared to controls (Chi2 = 8.1288; df = 3 p = 0.043). These results indicate that a high proportion of suicide completers (50% vs. 11.8% for controls) present *TrkB-T1* expression levels which correspond to the lowest extreme of the distribution.

The differences in *TrkB-T1* expression levels could not be explained by age (t = −1.548; df = 36 p = 0.130) pH (t = 0.634; df = 36 p = 0.53) PMI(t = 0.107; df = 36; p = 0.404) or suicide methods (t = 0.482; df = 36; p = 0.404).

### Analysis of hsa-miR185* Expression in the Replication Case-control Sample Confirmed an Inverse Correlation with *TrkB-T1* Expression

We next analyzed the expression level of Hsa-miR-185* in the sample of 55 individuals and found a significant increase in Hsa-miR-185* expression in the suicide completers (Figure 2C, t = 2.603; df = 53, p = 0.012) compared to controls. This increase was not explained either by differences in pH, age or PMI nor by psychopathology, course of illness, ante-mortem medication or substance use (p>0.05 for all factors; see Supporting information S1). Furthermore, in relating the levels of *TrkB-T1* levels to those of Hsa-miR-185* in the 55 patients investigated, we observed a significant inverse linear correlation between these two molecules (Figure 2D, n = 55 R = −0.439 p = 0.001) Pearson correlation.

No single individual or groups of individuals were driving the effect of the inverse correlation between Hsa-miR-185* and Trkb.T1. To demonstrate this idea, beside the correlation of the total samples, we performed a correlation analysis of baskets of the sample ranging from low to high expression (Total sample, rsq = 0.191 p = 0.001; 0–1.5 T1 expressors: rsq = 0.373 p = 0.003; 0–3 T1 expressors; rsq = 0.103 p = 0.04).

These results are not generalizable to all brain regions since no difference was observed in the cerebellum. Furthermore, no difference of TrkB full length protein levels was observed in BA10 (see Supporting information S1 and Figure S4). In addition, other microRNAs with putative binding on *TrkB-T1* did not show differences between groups (see Supporting information S1 and Figure S5).

*TrkB-T1* and Hsa-miR-185* expression levels were not associated with variation in TrkB-T1 3′UTR sequence or in the region containing DNA encoding Hsa-miR-185 microRNA (see Supporting information S1, Supporting Table S5 and Figure S3).

### Functional Analysis of Hsa-miR-185* on the Modulation of *TrkB-T1* Expression in Human Cell Lines

Multiple human cell lines were first assessed for expression of both Hsa-miR-185* and TrkB.T1. We found that both Hsa-miR-185* and *TrkB-T1* are relatively well expressed in the neuronal line CRL2137, so this cell line was used to analyze the effects of Hsa-miR-185* silencing on the modulation of *TrkB-T1* expression. The embryonic renal cell line HEK293 strongly expresses *TrkB-T1*, so this cell line was used to observe the effects of induced Hsa-miR-185* overexpression on the levels of *TrkB-T1* (Figure S2).

#### 1. Functional analysis of Hsa-miR-185* silencing on the modulation of TrkB-T1 expression

**Figure 3:**
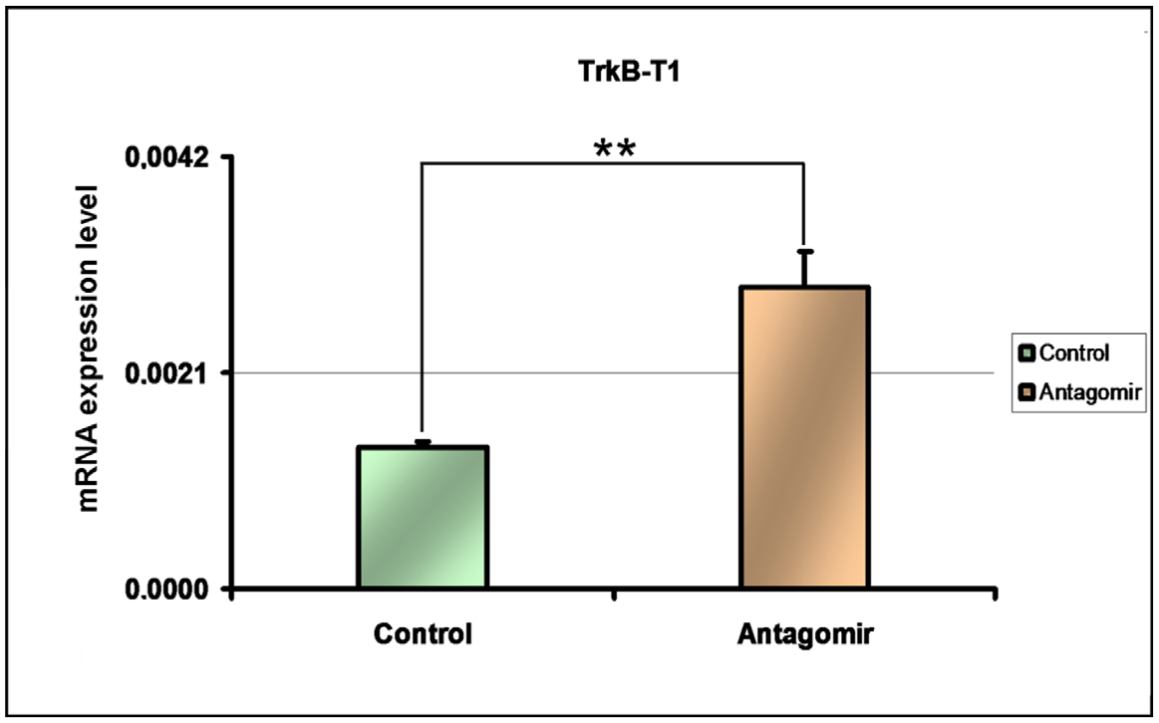
Effect of Hsa-miR-185* silencing on *TrkB-T1* expression in CRL 2137 cell line. Significant increase of *TrkB-T1* in cells transfected with antagomir for Hsa-miR-185* compared to the negative control. *: p<0.05 **: p<0.01 N_negativecontrol_ = 5 N _antagomir_ = 5 Error bar indicate standard errors of the mean. All statistical comparisons were done by t test.

CRL2137 cells from the neuronal line CRL2137 were cultured for a period of three hours and then transfected either with an antagomir that inhibits specifically Hsa-miR-185*, or alternatively, with a negative control not known to bind any mammalian RNA sequences. RNA was extracted following transfection and expression levels *TrkB-T1* were measured by RT-PCR. We observed a significant increase of *TrkB-T1* expression levels in the cells transfected by the Hsa-miR-185* antagomir (t = 5.054; df = 5, p = 0.0033) (Figure 3) compared to cells transfected with the negative control.

#### 2. Analysis of the functional effect of Hsa-miR-185* exogenous expression on the modulation of TrkB-T1 levels

**Figure 4:**
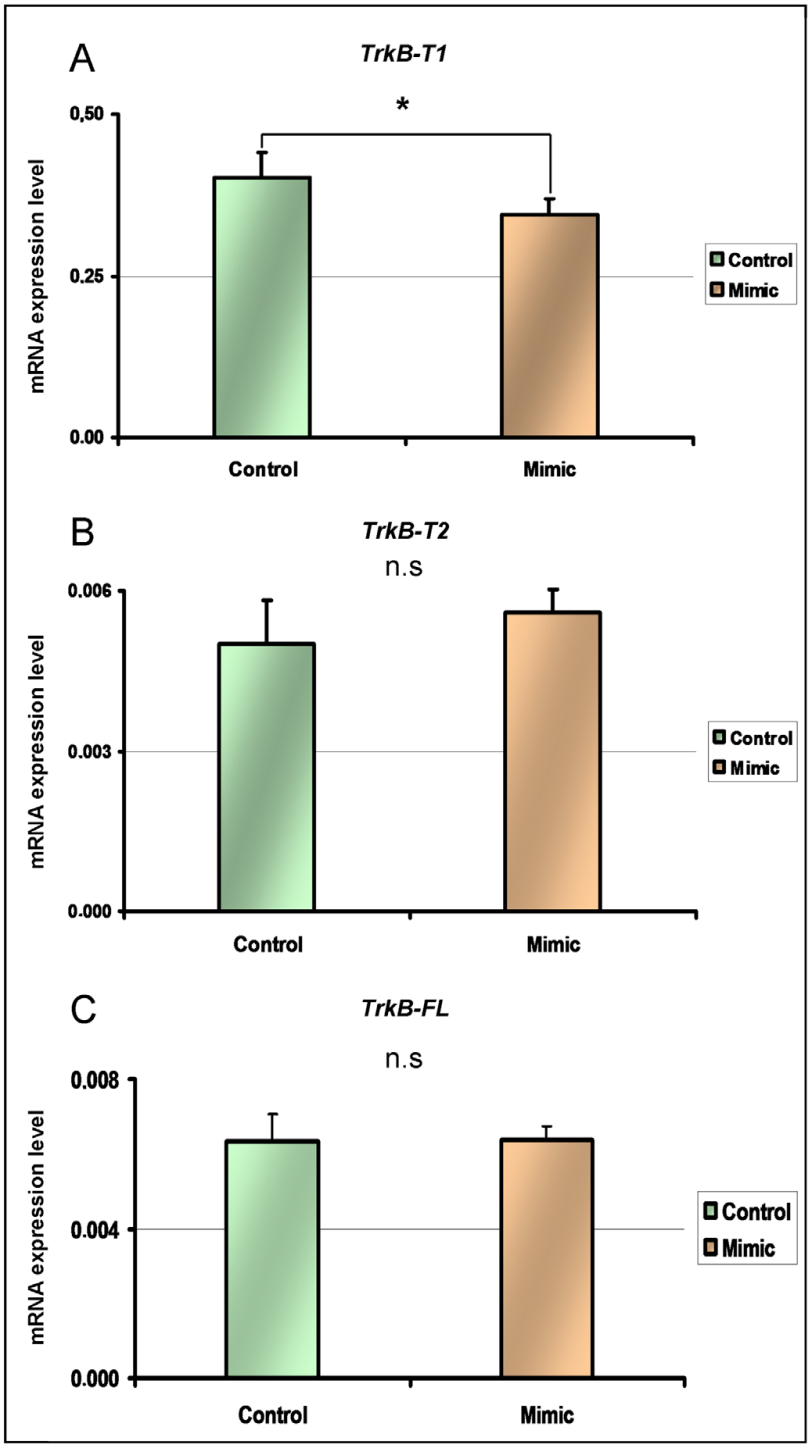
Effect of Hsa-miR-185* exogenous expression on TrkB-T1 levels in HEK293 cell line. A: Decrease of *TrkB-T1* in cells transfected with mimic compared to the negative control. B: No significant change of *TrkB-T2* expression levels in cells transfected with mimic compared to negative control. C: No significant change of *TrkB-FL* in cells transfected with mimic compared to negative control. *:p<0.05 n.s: not significant. N_negativecontrol_ = 5 N_mimic_ = 5 Error bars indicate standard errors of the mean. All statistical comparisons were done by t test.

In order to confirm that an interaction exists between microRNA Hsa-miR-185* and *TrkB-T1*, HEK293 cells, that strongly express TrkB-T1, were cultured for 24 hours and transfected with either an Hsa-miR-185* mimic, or negative control microRNA. Following transfection, RNA was extracted and expression levels of Hsa-miR-185* and *TrkB-T1* were quantified by RT-PCR. We observed a significant reduction of *TrkB-T1* expression levels in the cells transfected by the mimic compared to those transfected with the negative control (t  =  2; 885, df = 9, p = 0.018) (Figure 4A).

According to the NCBI database, in humans, there are 5 alternative transcripts of the gene that codes for TrkB (NTRK2), but only one 3′ UTR sequence specific to TrkB-T1. To ensure the specificity of Hsa-miR-185* on the 3′UTR sequence of the *TrkB-T1* transcript, we quantified the expression levels of the *TrkB-T2* and *TrkB-FL* transcripts in the HEK293 cells transfected by the Hsa-miR-185* mimic.No significant difference in the expression levels of TrkB-T2 and TrkB-FL was detected in the cells transfected by the Hsa-miR-185* mimetic when compared to cells transfected with a negative control. (Figure 4B and 4C).

#### 3. Functional analysis of the putative binding sites of Hsa-miR-185* in the 3′UTR sequence of *TrkB-T1*

Five putative binding sites for Hsa-miR-185* are present in the 5165 bp 3′UTR sequence of *TrkB-T1* (Figure S1). We cloned a 3.7 kb fragment of *TrkB-T1* 3′UTR sequence containing all five putative binding sites and co-transfected this construct with Hsa-miR-185* mimic or a negative control oligonucleotide in HEK293 cells. We observed a significant decrease of luciferase activity (7%) in HEK 293 cells transfected with mimic compared to cells transfected with a negative control oligonucleotide (t = 3.007; df = 10, p = 0.027).

**Figure 5:**
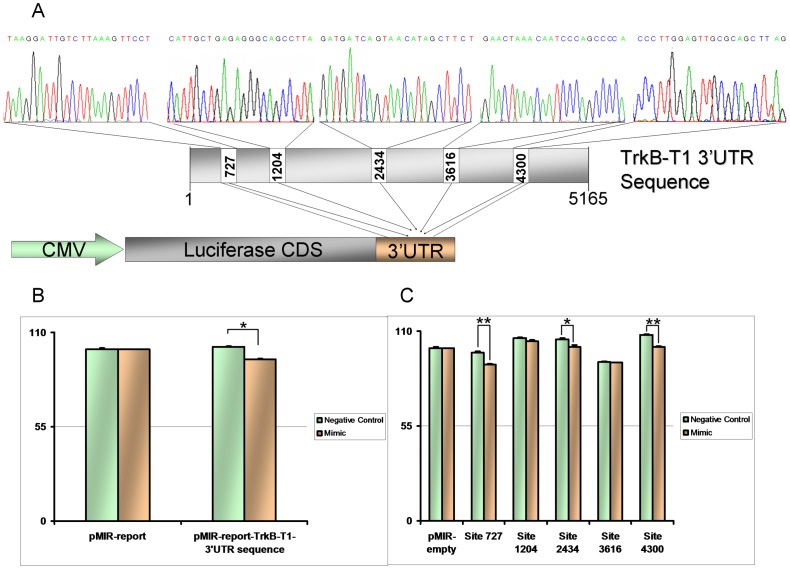
Functional effect of the Hsa-miR-185* on the *TrkB-T1* 3′UTR sequence in HEK 293 cell line. A: Schematic representation of *TrkB-T1* 3′UTR sequence including the five putative binding sites for Hsa-miR-185*. B: Significant decrease of luciferase activity quantified in cells transfected with 3′UTR sequence containing the five putative binding sites C: Luciferase activity quantified on cells transfected with five individual fragments of TrkB-T1 3′UTR sequence containing only one of the five putative binding sites. *: p<0.05 **: p<0.01 N_negative control_ = 6, N_mimic_ = 6. Error bars indicate standard errors of the mean. All statistical comparisons were done by t test.

In order to determine which of the five putative Hsa-miR-185* binding sites were contributing to the decrease in luciferase activity, we cloned each site in 130 to 330 bp fragments downstream to the luciferase coding sequence. These constructs were individually co-transfected into HEK293 cells with a Hsa-miR-185* mimic and a normalization vector in adherence to the conditions defined by Larsson et al. [34]. Compared to negative controls, we observed a decrease of 7% in luciferase activity in HEK293 cells transfected with Hsa-miR-185* mimic and containing putative binding site 727 (referring to the nucleotide position after the stop codon; t = 3.862; df = 9, p = 0.0038). In addition, a significant reduction of 4% (t = 2.591; df = 10, p = 0.027) was observed in HEK293 cells transfected with Hsa-miR-185* mimic and containing the putative binding site 2434 compared to cells transfected with the negative control. Finally, we observed a decrease of 6.6% in luciferase activity (t = 3.256; df = 10, p = 0.0086) in cells transfected with Hsa-miR-185* mimic and containing putative binding site 4300 (Figure 5). On the other hand, sites 1204 and 3616 showed no difference in luciferase activity when transfected with Hsa-miR-185* mimic and compared to negative controls.

## Discussion

In this study, we presented multiple lines of evidence suggesting a role for microRNA Hsa-miR-185* in the regulation of TrkB-T1, a truncated TrkB transcript whose downregulation has been associated with suicide. The expression level of this microRNA is significantly increased in frontal cortex from suicide completers and negatively correlated with *TrkB-T1* expression level. Through functional analyses using silencing or exogenous expression of Hsa-miR-185*, we confirmed that *TrkB-T1* expression levels can be regulated by this microRNA in vitro. Finally, we characterized five potential binding sites for Hsa-miR-185* in *TrkB-T1* 3′ UTR sequences, three of which are functional.

Since their discovery in C elegans in 1993 [35], small interfering RNA have instigated a great interest on behalf of the scientific community. Their involvement has been described in development [36], neurological diseases [37] and cancer [38], [39] among others. Their biological impact appears to be very important, and so these molecules provide avenues for new therapeutic interventions [40], [41], [42]. In spite of these attractive perspectives, little is known about the potential role of small noncoding RNA molecules in psychiatric disorders, and more specifically in depression and suicide. In this context, to our knowledge, our study provides the first evidence of microRNA involvement in the molecular mechanisms underlying suicide.

RNA interference is a process whereby organisms regulate mRNA through small noncoding RNAs, which are primarily of two types. The small interfering RNAs (siRNA) are RNA duplexes that induce sequence-specific gene silencing [43], and microRNA, which differently from siRNA, have incomplete base complementarity to their target sequences. Hsa-miR-185* was first identified in a human neuroblastoma cell line treated with retinoic acid [44] and its locus maps to the velocardiofacial syndrome region on 22q11.2, which has been of significant interest to psychiatry because it is the region deleted in Velocardiofacial/DiGeorge’s syndrome, where ARVCF, COMT and DGCR8 genes are located. Duplications and deletions in this region have been consistently associated with schizophrenia [45], [46]. The current study suggests that deletions or duplications in Hsa-miR-185* could potentially impact expression levels of TrkB-T1 and lead to a psychiatric phenotype. A murine model of the 22q11.2 deletion has recently been developed and, among other findings, these mice present morphological abnormalities in dendritic spines at glutamatergic synapses [47] and also anomalous maturation of microRNAs [48]. Our results suggest that the 22q11.2 region is an interesting candidate region to consider when conducting functional analysis of microRNAs investigating the neurobiology of suicide and related phenotypes.

Hsa-miR-185* is the complementary microRNA strand form (frequently referred to as the “passenger strand”) associated with Hsa-miR-185. Two consecutive cleavages involving Drosha/DGCR8 and TRBP/Dicer complexes provide primicroRNA transcribed from genomic DNA to a RNA/RNA duplex [49] containing the leader strand, which targets mRNA, and the passenger strand (3p or* form), which is frequently – but not always – destroyed by the cells. Accordingly, the passenger form of microRNA can also have an inhibitory activity [50]. The functional role of the microRNA* strand has been well demonstrated and its implication in pathophysiological mechanisms has already been reported by several groups. Jadzewski and collaborators [51] have shown an increase in the different alternative forms of pre-miR-146a in thyroid cancer. These microRNA variants contain a polymorphism which determines the repertoire of potential targets, some of which are implicated in apoptosis and DNA repair [51]. Although most microRNA studies have focused on the guide strand, a growing number of investigations have shown that the passenger strand can also have an effect of transcriptional or post-transcriptional regulation. For example, modifications to the passenger strand of miR-30 have been noted to affect the induction of interferon and diminish the immune response involved in cellular mediation [52]. This immune response, associated with increased interferon expression, has been shown to affect the shape dendritic spines and of synapses [53], [54].

By focusing on individuals with extreme low *TrkB-T1* levels, our study was designed to identify microRNAs that may regulate TrkB-T1 expression, and our results suggest that Hsa-miR-185* may play such a role; however, it is unclear why expression of Hsa-miR-185* is increased. In the current study, subjects were not assessed for micro duplications at the DiGeorge region, but this may be a worthwhile test to undertake, as this microRNA is expressed from DiGeorge critical region on chromosome 22. In addition to variation in the genomic sequences coding for microRNAs, dysregulation may be explained by a number of different factors, including changes in levels of proteins that play a role in the maturation process of microRNA. An increase of DGCR8, another gene in the DiGeorge ciritical region and which contributes to primiRNA maturation, is responsible for microRNA dysregulation in cortical regions from schizophrenic patients [55]. Similarly, changes in levels of proteins involved in the processing of microRNA passenger strand, such as AGO1 and AGO2 [56], may regulate expression levels of this form of microRNAs. Although microRNA biogenesis [57] and subsequent alteration has been associated with pathological conditions [58], further investigation is needed in order to better understand the maturation process of the passenger strand.

We performed functional assays where several putative TrkB-T1 3′UTR. Hsa-mIR- 185* binding targets were cloned into a luciferase vector to determine if we could experimentally decrease luciferase activity by co-transfection with an Hsa-mir-185* mimic. The luciferase assays suggested that Hsa-miR-185* might induce a small decrease of TrkB-T1 expression by binding 3′UTR sites, given the subtle but significant decreases in luciferase activity. Several factors could affect the interpretation. We used HEK293 cells, where we detected 10-fold higher levels of TrkB-T1 expression compared to the neuronal cell line we used. While the choice of HEK293 cells may have been more appropriate than the neuronal cell line – any cell type that expresses modest levels of TrkB-T1 may also be expected to have relative levels of regulatory elements that modify the expression of TrkB-T1– reproducing this experiment in multiple cell lines known to express TrkB-T1 are warranted. The very subtle decrease also needs to be assessed systematically where the dynamic range of the assay can be measured; specifically, small decreases need to be detectable above basal ‘noise’ levels across replicates. Finally, the basic biology of mood disorders is not necessarily expected to produce large, high penetrance effects. The physiological implication of modest down-regulation of a particular gene is not clear – a slight decrease in expression could lead to psychiatric pathology. Even in non-psychiatry fields, several other functional studies have also shown moderate effects of microRNA on luciferase activity [59], [60], [61], [62], [63], suggesting that our data, and microRNA regulation more generally, produce subtle alterations in gene product.

The interaction properties of a microRNA with a target mRNA is fundamental to final expression levels of the mRNA. The potential effect of a microRNA on its target depends on the strength of the interaction between the microRNA sequence and the 3′UTR sequence of the mRNA. This interaction depends on the complementarity between the two sequences and is characterized by folding energy or energy required to disrupt the interaction. The folding energy of the interaction between Hsa-miR-185* and the *TrkB-T1* 3′UTR is estimated to be approximately −25 kj/mol, an amount similar to the estimated folding energies of other microRNA and 3′UTR sequence interactions investigated (for instance, Hsa-miR-145 and STAT1 [60] or Hsa-miR-659 and GRN [62]), which also show subtle effects. Hsa-miR-185* could also be rapidly degraded after cleavage by the TRBP/dicer complex [22]. Even with efficient binding to the mRNA, this could lead to an equilibrium where very little mRNA is degraded Finally, some RNA binding proteins such as ELAVL1 or PABPC1 are known to be expressed in HEK293 cells [64], [65], [66] and may bind TrkB mRNA [67]. The expression of these genes and their binding activity might interfere with the interaction between Hsa-miR-185* and TrkB-T1 [68] and partially contribute to the attenuate the functional effect of The Hsa-miR-185* on TrkB-T1 observed in HEK293 cells.

In previous studies, we have shown that other molecular mechanisms, such as site specific methylation of the promoter sequence of the NTRK2 gene [12] and the methylation of the lysine 27 residue of histone H3 [19], contribute to a reduction of the *TrkB-T1* transcript expression. However, neither is sufficient to explain the levels of downregulation of TrkB-T1 observed in suicide. In the current study, we have shown the functional impact of the up-regulation of Hsa-miR-185* on the reduction of TrkB-T1 expression levels. Together, these results may suggest that the reduction of TrkB-T1 in frontal cortex of suicide completers results from the combined effects of altered histone methylation, decreased promoter methylation, and increased expression of of Hsa-miR-185*.

This study is not without limitations. We used a microarray approach with a preselected sample based on *TrkB-T1* expression levels. Because the sample size used for this experiment was small, we have probably only identified a limited amount of the variability in microRNA’s that might regulate *TrkB-T1*, a hypothesis consistent with *in silico* predictions of interactions between microRNAs and the *TrkB-T1* 3′UTR sequence. Despite the limited power of our screening sample, our findings were reproduced in a larger independent sample set where we performed a targeted RT-PCR screen of Hsa-mir 185* expression in human frontal cortex. Our study also does not investigate the mechanisms underlying changes in microRNA expression. Specifically, we performed correlative experiments to demonstrate that Hsa-mir 185* has an effect on TrkB-T1 expression, but we did not investigate why Hsa-miR-185* is decreased in the first place.

## Supporting Information

Figure S1 Interaction between *TrkB-T1* 3′UTR sequence and Hsa-miR-185* predicited by RNA22 software.
(TIF)

Figure S2 Basal expression levels of Hsa-miR-185* and *TrkB-T1* in CRL2137 and HEK293 cells N = 3 for each condition.
(TIF)

Figure S3 Analyses of Hsa-miR-185 region by sequencing on the independent replication sample composed of 17 controls and 38 suicide completers. A: Schematic representation of SNPs found in a 1.6 kb sequence comprising the 82 sequencing coding for Hsa-mir-185, 1084 bases in the upstream and 516 in the downstream region. Two tag SNPs identified: rs2078749 and rs2008591. B: Statistics summarizing the tests for an allelic association between polymorphisms at tSNPs and suicide. C: Statistics summarizing the tests a haplotypic association involving polymorphisms at tSNPs and suicide. D: graph bar of the Hsa-miR-185* expression level depending on the genotypes at tSNP rs2078749. E: graph bar of the Hsa-miR-185* expression level depending on the genotypes at tSNP rs2008591.
(TIF)

Figure S4 Graph bar of the quantification of TrkB-FL isoform performed by western blot on the screening sample composed of suicide completers selected according to extreme low *TrkB-T1* levels and controls with normal *TrkB-T1* expression values (N = 8). Comparison was done by t test.
(TIF)

Figure S5 Graph bar of the quantification of microRNAs that could bind *TrkB-T1* 3′UTR sequence in our cohort of 55 patients. A: Graph bar of the quantification of Hsa-miR-423-5p. B: Graph bar of the quantification of Hsa-miR-193a-5p. All statistical comparisons were done by t test.
(TIF)

Table S1 Primers used to generate and clone fragments of *TrkB-T1* 3′UTR sequence in pMIR report vector.
(DOC)

Table S2 Age, pH and PMI of matched controls and suicide completers included in the microarray study.
(DOC)

Table S3 List of microRNA probesets expressed in BA10 with average group expression values.
(DOC)

Table S4 Mean age, pH and PMI ± standard deviation for the independent larger sample of 55 individuals (17 controls and 38 suicide completers) investigated in the replication study.
(DOC)

Table S5 Statistics describing tests for allelic assciations between SNPs located in the fragment containing site 727 from *TrkB-T1* 3′UTR sequence, and suicide.
(DOC)

Supporting Information S1 Supporting Methods, Supporting Results, Supporting References.
(DOC)
