# Supplementary figures and images for: Regulation of a Truncated Form of Tropomyosin-Related Kinase B (TrkB) by Hsa-miR-185* in Frontal Cortex of Suicide Completers
Source: PLoS One. 2012 Jun 25;7(6):e39301. doi: 10.1371/journal.pone.0039301 (PMC3382618; doi:10.1371/journal.pone.0039301)

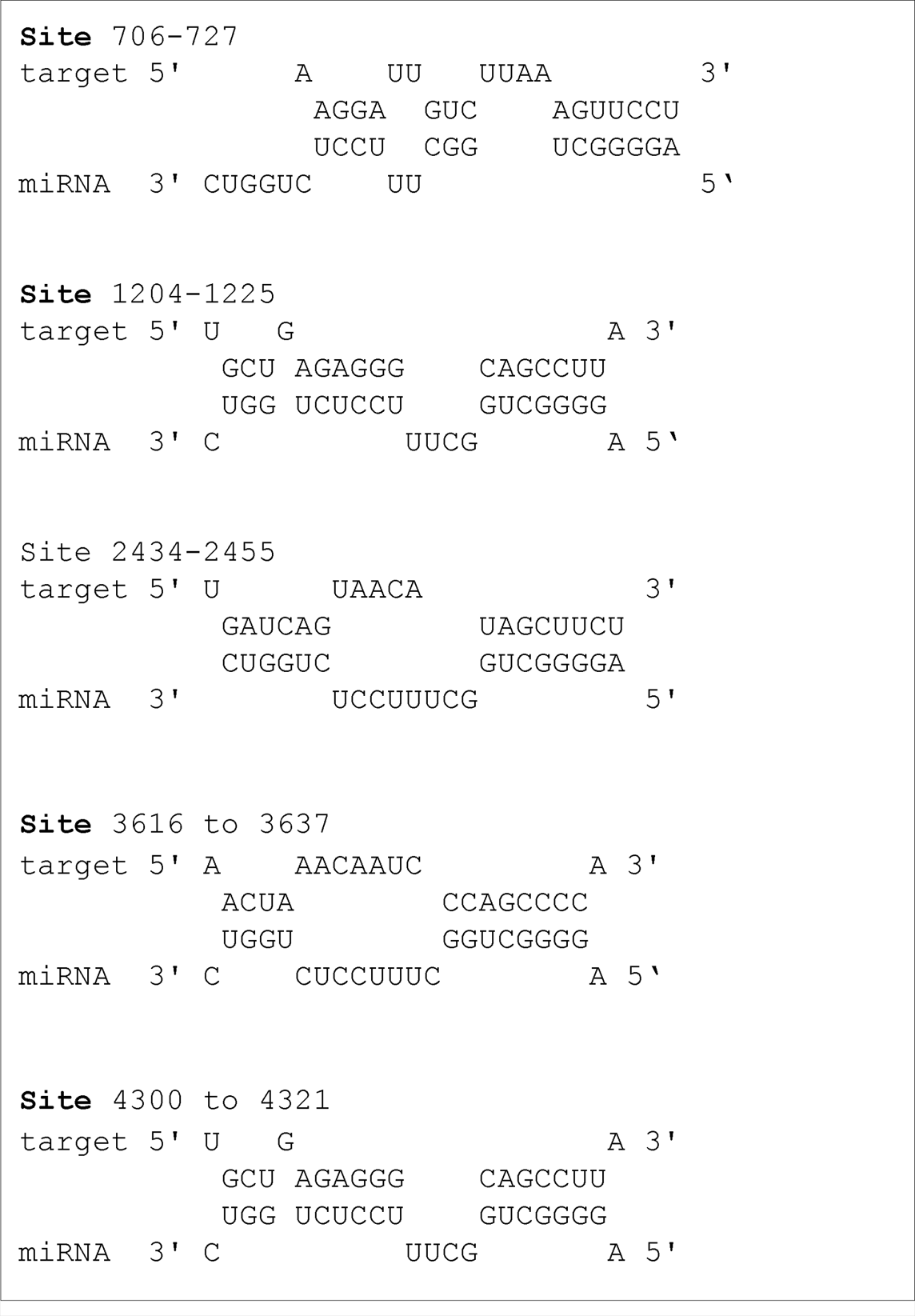

Supplement: Figure S1 — Interaction between TrkB-T1 3′UTR sequence and Hsa-miR-185* predicited by RNA22 software. (TIF) [file pone.0039301.s001.tif]

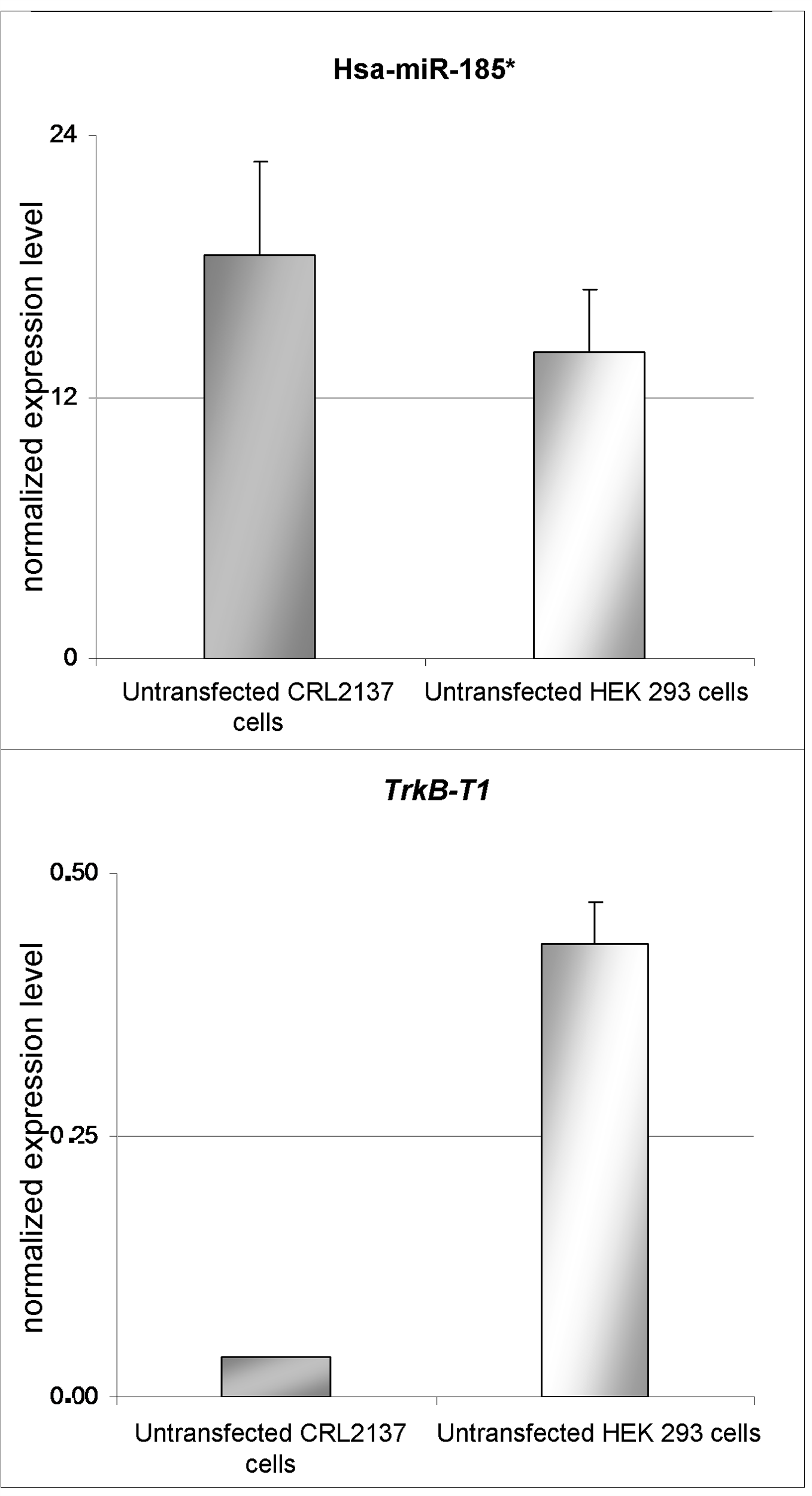

Supplement: Figure S2 — Basal expression levels of Hsa-miR-185* and TrkB-T1 in CRL2137 and HEK293 cells N = 3 for each condition. (TIF) [file pone.0039301.s002.tif]

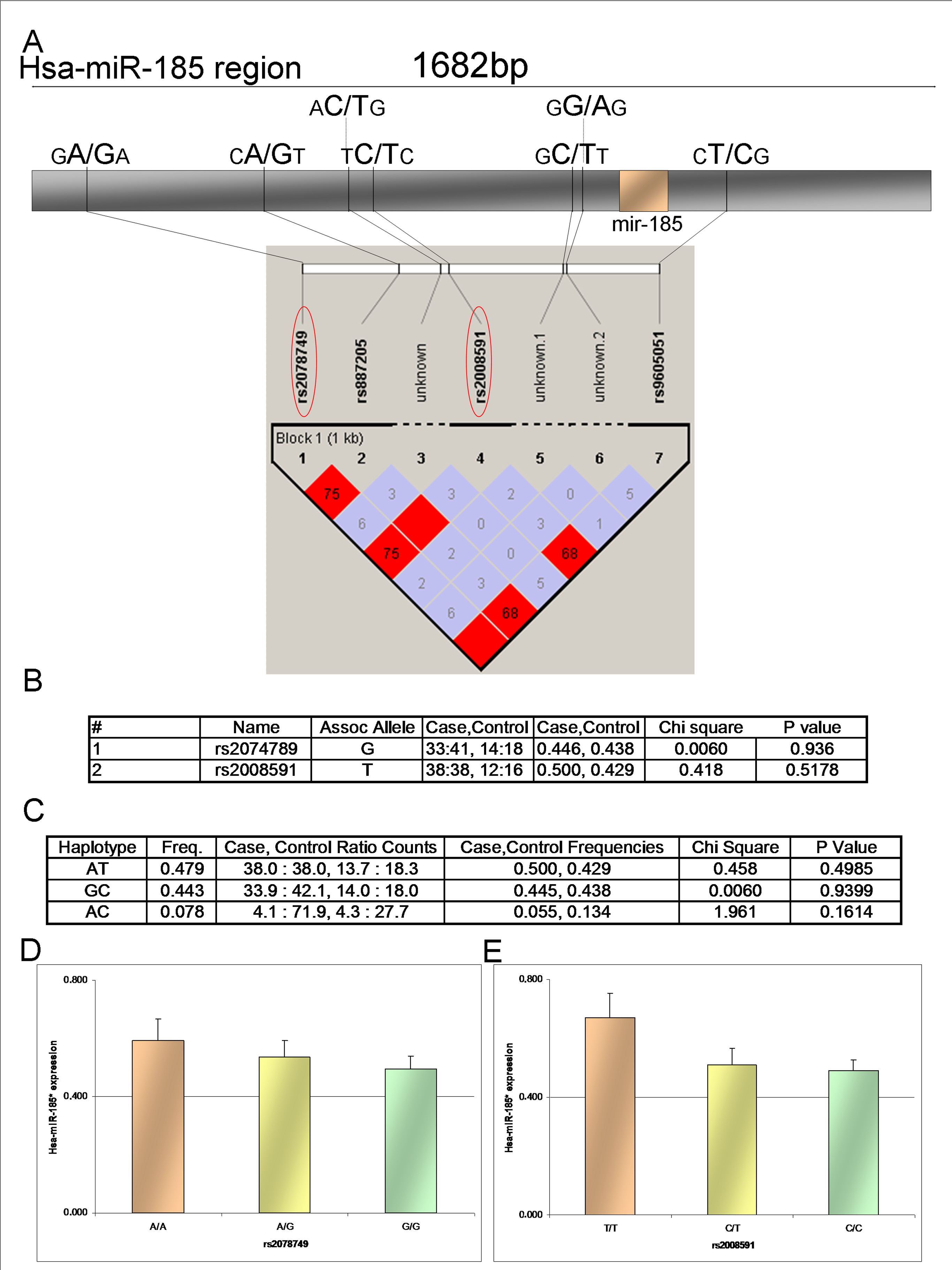

Supplement: Figure S3 — Analyses of Hsa-miR-185 region by sequencing on the independent replication sample composed of 17 controls and 38 suicide completers. A: Schematic representation of SNPs found in a 1.6 kb sequence comprising the 82 sequencing coding for Hsa-mir-185, 1084 bases in the upstream and 516 in the downstream region. Two tag SNPs identified: rs2078749 and rs2008591. B: Statistics summarizing the tests for an allelic association between polymorphisms at tSNPs and suicide. C: Statistics summarizing the tests a haplotypic association involving polymorphisms at tSNPs and suicide. D: graph bar of the Hsa-miR-185* expression level depending on the genotypes at tSNP rs2078749. E: graph bar of the Hsa-miR-185* expression level depending on the genotypes at tSNP rs2008591. (TIF) [file pone.0039301.s003.tif]

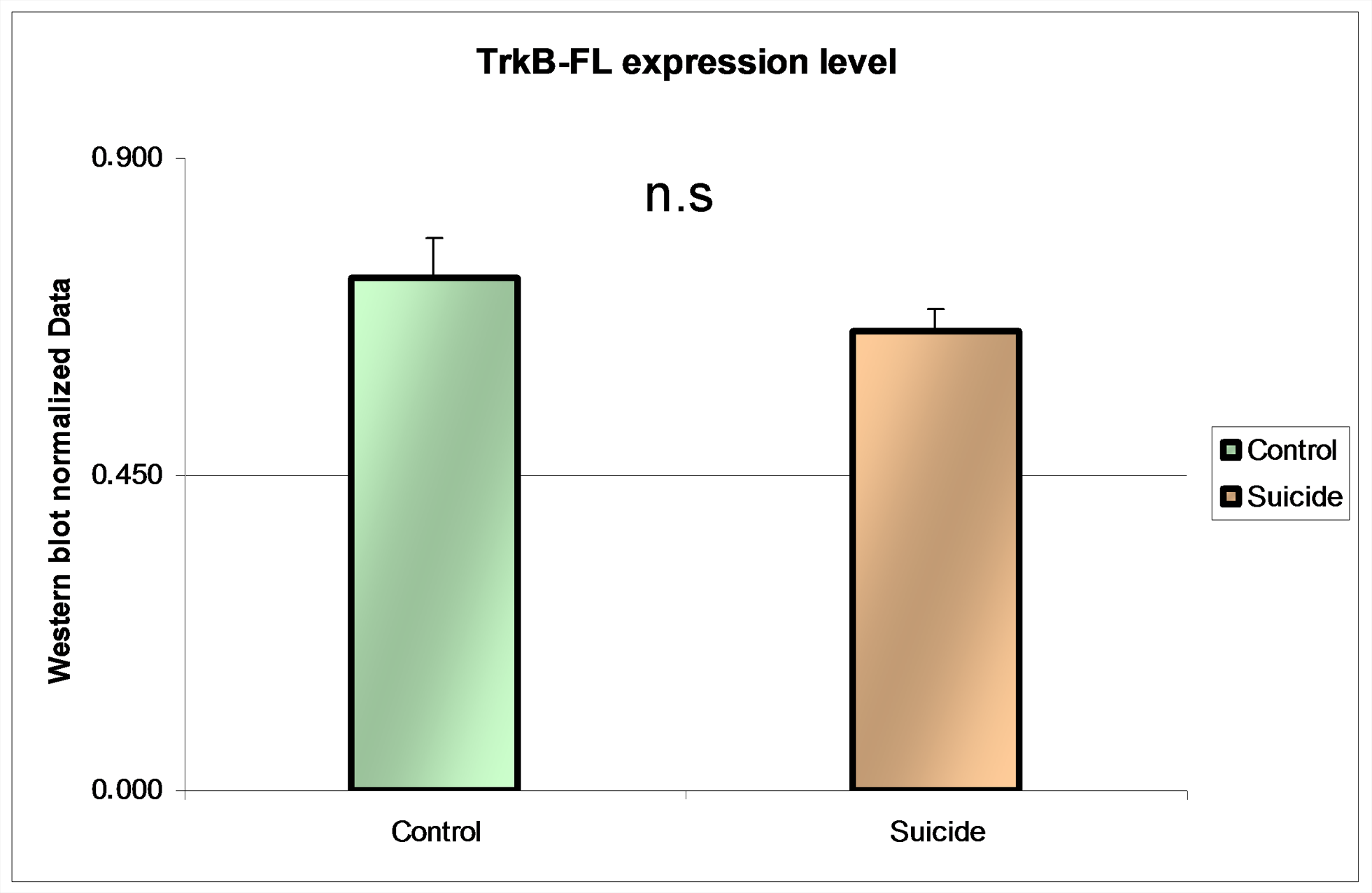

Supplement: Figure S4 — Graph bar of the quantification of TrkB-FL isoform performed by western blot on the screening sample composed of suicide completers selected according to extreme low TrkB-T1 levels and controls with normal TrkB-T1 expression values (N = 8). Comparison was done by t test. (TIF) [file pone.0039301.s004.tif]

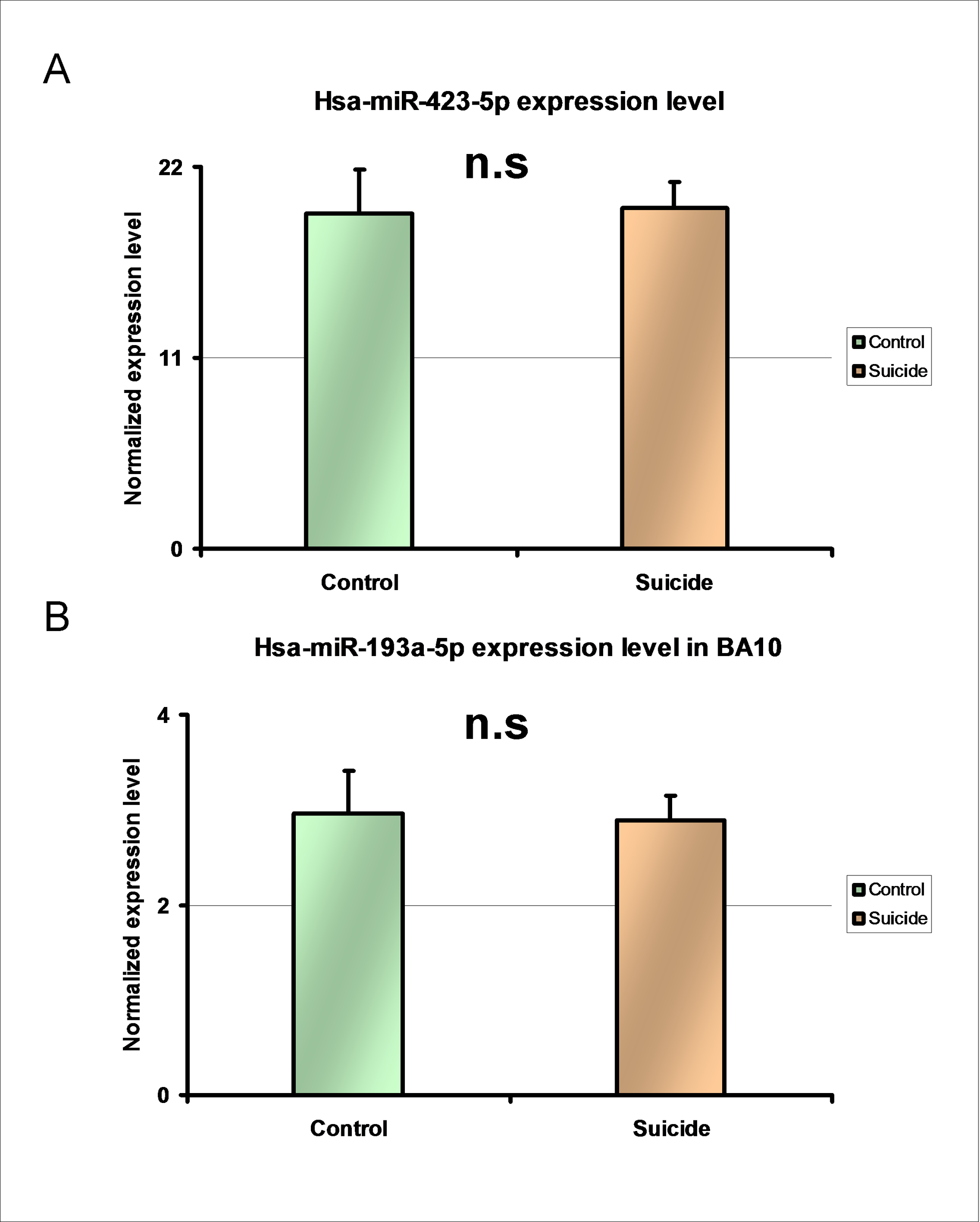

Supplement: Figure S5 — Graph bar of the quantification of microRNAs that could bind TrkB-T1 3′UTR sequence in our cohort of 55 patients. A: Graph bar of the quantification of Hsa-miR-423-5p. B: Graph bar of the quantification of Hsa-miR-193a-5p. All statistical comparisons were done by t test. (TIF) [file pone.0039301.s005.tif]
