# Supplementary material for: Regulation of a Truncated Form of Tropomyosin-Related Kinase B (TrkB) by Hsa-miR-185* in Frontal Cortex of Suicide Completers
Source: PLoS One. 2012 Jun 25;7(6):e39301. doi: 10.1371/journal.pone.0039301 (PMC3382618; doi:10.1371/journal.pone.0039301)
Supplement: Table S1 — Primers used to generate and clone fragments of TrkB-T1 3′UTR sequence in pMIR report vector. (DOC) [file pone.0039301.s006.doc]

Supporting Table S1

Table S1A

| Fragments | Primer Up | Primer Dw |
| --- | --- | --- |
| Site 727  (209bp) | GGACTTGGTAGTATTATTAAAAGGTTA | CAACAAAACAGCTGTTGCTGC |
| Site 1204 (260bp) | GAGCAGGAGAGGAGATTCTA | CAGAGCATACAAAGGTCACT |
| Site 2434 (181bp) | GAATGATGAAAACAGCAGGCTATTAG | GGAAGCTCTAAAAAGCACATTTCTTA |
| Site 3616 (130bp) | AGGGACTGATTTCATTTCTGACC | GGAGAGTGAGAAGCCAAAAGCAA |
| Site 4300 (143bp) | GTTGGTGCATCCAGCCACAT | AACATAGCAGAACACACTTAATA |
| TrkB-T1 3’UTR 3.7kb | GGACTTGGTAGTATTATTAAAAGGTTA | AACATAGCAGAACACACTTAATA |

Table S1B

| Fragments | Primer | Sequence |
| --- | --- | --- |
| Site 727 | Up | TAATACTAGTGGACTTGGTAGTATTATTAAAAGGTTA |
|  | Dw | TAATAAGCTTCAACAAAACAGCTGTTGCTGC |
| Site 1204 | Up | TAATGAGCTCGAGCAGGAGAGGAGATTCTA |
|  | Dw | TAATACGCGTCAGAGCATACAAAGGTCACTG |
| Site 2434 | Up | TAATACTAGTGAATGATGAAAACAGCAGGCTATTAG |
|  | Dw | TAATAAGCTTGGAAGCTCTAAAAAGCACATTTCTTA |
| Site 3616 | Up | TAATACTAGTAGGGACTGATTTCATTTCTGACC |
|  | Dw | TAATAAGCTTGGAGAGTGAGAAGCCAAAAGCAA |
| Site 4300 | Up | TAATACTAGTGTTGGTGCATCCAGCCACAT |
|  | Dw | TAATAAGCTTAACATAGCAGAACACACTTAATA |
| TrkB-T1 3’UTR 3.7kb | Up | TAATACGCGTGGACTTGGTAGTATTATTAAAAGGTTA |
|  | Dw | TAATACGCGTAACATAGCAGAACACACTTAATA |
