# Supplementary material for: Regulation of a Truncated Form of Tropomyosin-Related Kinase B (TrkB) by Hsa-miR-185* in Frontal Cortex of Suicide Completers
Source: PLoS One. 2012 Jun 25;7(6):e39301. doi: 10.1371/journal.pone.0039301 (PMC3382618; doi:10.1371/journal.pone.0039301)
Supplement: Table S2 — Age, pH and PMI of matched controls and suicide completers included in the microarray study. (DOC) [file pone.0039301.s007.doc]

Supporting Table S2

|  |  | Age | pH | PMI |
| --- | --- | --- | --- | --- |
| Control | Mean | 36 | 6,53 | 23,25 |
| n=4 | Stand. Dev | 7 | 0,05 | 4,62 |
| Suicide | Mean | 31 | 6,62 | 26,88 |
| n=4 | Stand. Dev | 4 | 0,02 | 2,70 |
