# Supplementary material for: Regulation of a Truncated Form of Tropomyosin-Related Kinase B (TrkB) by Hsa-miR-185* in Frontal Cortex of Suicide Completers
Source: PLoS One. 2012 Jun 25;7(6):e39301. doi: 10.1371/journal.pone.0039301 (PMC3382618; doi:10.1371/journal.pone.0039301)
Supplement: Table S3 — List of microRNA probesets expressed in BA10 with average group expression values. (DOC) [file pone.0039301.s008.doc]

Supporting Table S3: List of microRNA probesets expressed in BA10 with average group expression values:

| Annotation | Column ID | T-test | Mean log | Standard error | Mean log ratio | Standard error | Mean Log ratio | Foldchange | Variation | A Mean |  |
| --- | --- | --- | --- | --- | --- | --- | --- | --- | --- | --- | --- |
|  | Probe Id |  | Control |  | Suicide |  |  | Suicide vs Contrôle |  |  |  |
| hsa-miR-491-3p | 17927 | 0,00009 | -0,40 | 0,05 | 0,12 | 0,02 | 0,52 | 1,44 | up | 11,10 | * |
| hsa-miR-185* | 17904 | 0,00018 | -1,03 | 0,15 | 0,68 | 0,14 | 1,72 | 3,28 | up | 10,69 | * |
| hsa-miR-888* | 42825 | 0,00113 | -0,16 | 0,03 | -0,45 | 0,04 | -0,29 | -1,22 | dw | 10,57 |  |
| hsa-miR-886-5p | 17885 | 0,00288 | 0,33 | 0,08 | -0,30 | 0,10 | -0,62 | -1,54 | dw | 8,80 |  |
| hsa-miR-125b-2* | 42845 | 0,00323 | -0,21 | 0,04 | 0,06 | 0,04 | 0,27 | 1,21 | up | 7,02 |  |
| hsa-miR-637 | 17354 | 0,01313 | -0,50 | 0,21 | 0,30 | 0,10 | 0,81 | 1,75 | up | 9,59 |  |
| hsa-miR-220b | 42900 | 0,01547 | 0,24 | 0,07 | -0,11 | 0,08 | -0,35 | -1,28 | dw | 7,80 |  |
| hsa-miR-551b | 42917 | 0,02351 | 0,08 | 0,04 | -0,51 | 0,19 | -0,59 | -1,51 | dw | 11,77 |  |
| hsa-miR-720 | 42751 | 0,03430 | -0,31 | 0,03 | -0,04 | 0,10 | 0,27 | 1,21 | up | 14,95 |  |
| hsa-miR-423-5p | 27565 | 0,04656 | 0,00 | 0,03 | 0,13 | 0,05 | 0,13 | 1,10 | up | 10,20 |  |
| hsa-miR-519d | 11163 | 0,04689 | -0,04 | 0,16 | -0,55 | 0,13 | -0,51 | -1,43 | dw | 11,96 |  |
| hsa-miR-125b-1* | 17876 | 0,04885 | 0,11 | 0,05 | 0,40 | 0,11 | 0,30 | 1,23 | up | 7,44 |  |
| hsa-miR-937 | 42514 | 0,05454 | -0,74 | 0,14 | -0,34 | 0,10 | 0,40 | 1,32 | up | 7,22 |  |
| hsa-miR-330-3p | 11063 | 0,05613 | -0,31 | 0,11 | -0,04 | 0,02 | 0,27 | 1,21 | up | 7,04 |  |
| hsa-miR-602 | 42531 | 0,05871 | -0,51 | 0,12 | -0,05 | 0,16 | 0,46 | 1,38 | up | 7,24 |  |
| hsa-miR-7-2* | 42964 | 0,06564 | 0,04 | 0,07 | -0,19 | 0,08 | -0,24 | -1,18 | dw | 7,17 |  |
| hsa-miR-642 | 42679 | 0,06735 | -0,38 | 0,26 | 0,24 | 0,10 | 0,62 | 1,53 | up | 11,72 |  |
| hsa-miR-302c* | 11045 | 0,06827 | 0,01 | 0,06 | -0,15 | 0,04 | -0,16 | -1,12 | dw | 7,52 |  |
| hsa-miR-361-3p | 42577 | 0,06969 | 0,31 | 0,05 | 0,42 | 0,02 | 0,11 | 1,08 | up | 6,83 |  |
| hsa-miR-149* | 42486 | 0,07921 | 0,09 | 0,04 | 0,24 | 0,06 | 0,15 | 1,11 | up | 10,26 |  |
| hsa-miR-620 | 32825 | 0,09277 | 0,11 | 0,15 | -0,21 | 0,06 | -0,33 | -1,25 | dw | 9,72 |  |
| hsa-miR-887 | 42711 | 0,09843 | 0,00 | 0,18 | -0,39 | 0,07 | -0,39 | -1,31 | dw | 6,83 |  |
| hsa-miR-525-5p | 11175 | 0,09966 | -0,22 | 0,09 | 0,00 | 0,07 | 0,22 | 1,17 | up | 8,52 |  |
| miRPlus_27560 | 27560 | 0,10313 | -0,42 | 0,22 | 0,16 | 0,21 | 0,58 | 1,50 | up | 9,48 |  |
| hsa-miR-513a-5p | 42581 | 0,11526 | -0,21 | 0,12 | 0,15 | 0,15 | 0,36 | 1,28 | up | 8,30 |  |
| hsa-miR-339-5p | 42739 | 0,12843 | -0,10 | 0,14 | 0,20 | 0,09 | 0,30 | 1,23 | up | 7,63 |  |
| hsa-miR-181a | 42865 | 0,13564 | -0,21 | 0,15 | 0,13 | 0,13 | 0,34 | 1,27 | up | 11,08 |  |
| hsa-miR-647 | 42746 | 0,13629 | 0,00 | 0,18 | -0,35 | 0,09 | -0,35 | -1,27 | dw | 6,75 |  |
| miRPlus_42856 | 42856 | 0,14533 | -0,07 | 0,09 | 0,12 | 0,07 | 0,19 | 1,14 | up | 10,41 |  |
| hsa-miR-193a-5p | 17878 | 0,15057 | -0,26 | 0,26 | 0,29 | 0,20 | 0,54 | 1,46 | up | 11,80 |  |
| hsa-miR-148b | 19585 | 0,15386 | -0,09 | 0,16 | 0,18 | 0,03 | 0,27 | 1,20 | up | 9,35 |  |
| hsa-miR-19a | 10997 | 0,15693 | 0,01 | 0,18 | 0,35 | 0,09 | 0,33 | 1,26 | up | 9,00 |  |
| hsa-miR-922 | 42683 | 0,15898 | 0,06 | 0,19 | -0,25 | 0,03 | -0,31 | -1,24 | dw | 7,05 |  |
| miRPlus_42780 | 42780 | 0,17362 | -0,27 | 0,24 | 0,15 | 0,12 | 0,42 | 1,34 | up | 8,02 |  |
| Annotation | Column ID | T-test | Mean log | Standard error | Mean log ratio | Standard error | Mean Log ratio | Foldchange | Variation | A Mean |  |
|  | Probe Id |  | Control |  | Suicide |  |  | Suicide vs Contrôle |  |  |  |
| hsa-miR-25 | 42682 | 0,17639 | -0,04 | 0,16 | 0,28 | 0,13 | 0,32 | 1,25 | up | 6,90 |  |
| hsa-miR-656 | 29736 | 0,18419 | 0,15 | 0,06 | -0,11 | 0,16 | -0,26 | -1,20 | dw | 7,12 |  |
| hsa-miR-768-5p | 29871 | 0,20435 | 0,01 | 0,11 | 0,21 | 0,10 | 0,21 | 1,15 | up | 10,39 |  |
| miRPlus_28302 | 28302 | 0,20647 | -0,44 | 0,06 | -0,25 | 0,12 | 0,19 | 1,14 | up | 9,28 |  |
| hsa-miR-330-5p | 42875 | 0,21112 | 0,10 | 0,06 | 0,23 | 0,06 | 0,13 | 1,09 | up | 7,49 |  |
| miRPlus_11239 | 11239 | 0,21642 | -0,03 | 0,02 | 0,05 | 0,05 | 0,08 | 1,05 | up | 7,68 |  |
| hsa-miR-337-3p | 42673 | 0,21731 | 0,02 | 0,06 | -0,12 | 0,08 | -0,14 | -1,10 | dw | 7,05 |  |
| hsa-miR-625* | 42699 | 0,22090 | 0,26 | 0,10 | 0,08 | 0,08 | -0,17 | -1,13 | dw | 9,28 |  |
| hsa-miR-338-5p | 17825 | 0,22131 | -0,19 | 0,19 | 0,26 | 0,27 | 0,45 | 1,37 | up | 8,83 |  |
| hsa-miR-18b | 42644 | 0,22471 | -0,10 | 0,12 | 0,15 | 0,14 | 0,25 | 1,19 | up | 7,05 |  |
| hsa-miR-628-5p | 17870 | 0,23154 | 0,37 | 0,05 | -0,12 | 0,37 | -0,49 | -1,41 | dw | 7,42 |  |
| hsa-miR-99b* | 17898 | 0,23453 | 0,05 | 0,04 | 0,12 | 0,03 | 0,07 | 1,05 | up | 7,78 |  |
| hsa-miR-106b | 19582 | 0,23467 | -0,08 | 0,12 | 0,09 | 0,04 | 0,17 | 1,12 | up | 10,66 |  |
| hsa-let-7b | 29502 | 0,23573 | -0,02 | 0,11 | 0,15 | 0,07 | 0,17 | 1,13 | up | 12,46 |  |
| hsa-miR-155 | 10964 | 0,23576 | 0,12 | 0,09 | 0,41 | 0,20 | 0,28 | 1,22 | up | 7,16 |  |
| hsa-miR-338-3p | 42592 | 0,23921 | -0,55 | 0,21 | -0,06 | 0,32 | 0,50 | 1,41 | up | 10,49 |  |
| *hsa-miR-185* | *42902* | *0,24156* | *-0,06* | *0,15* | *0,16* | *0,08* | *0,21* | *1,16* | *up* | *10,26* |  |
| hsa-miR-138-1* | 42872 | 0,24581 | -0,06 | 0,07 | -0,29 | 0,16 | -0,23 | -1,17 | dw | 9,77 |  |
| hsa-miR-18a | 42588 | 0,25053 | -0,13 | 0,11 | 0,09 | 0,14 | 0,23 | 1,17 | up | 7,16 |  |
| hsa-miR-550 | 42914 | 0,25391 | 0,11 | 0,06 | 0,00 | 0,07 | -0,11 | -1,08 | dw | 10,44 |  |
| hsa-miR-19b | 10998 | 0,25730 | 0,02 | 0,20 | 0,28 | 0,07 | 0,26 | 1,20 | up | 9,14 |  |
| hsa-miR-485-3p | 42694 | 0,25848 | 0,00 | 0,06 | -0,09 | 0,03 | -0,09 | -1,06 | dw | 9,49 |  |
| miRPlus_42530 | 42530 | 0,26020 | -0,27 | 0,18 | -0,51 | 0,06 | -0,24 | -1,18 | dw | 7,71 |  |
| hsa-miR-219-5p | 42509 | 0,27227 | -0,88 | 0,33 | -0,12 | 0,54 | 0,76 | 1,70 | up | 12,31 |  |
| hsa-miR-381 | 14306 | 0,27533 | -0,13 | 0,03 | -0,06 | 0,05 | 0,07 | 1,05 | up | 9,12 |  |
| hsa-miR-943 | 42696 | 0,27657 | -0,13 | 0,24 | 0,25 | 0,21 | 0,38 | 1,30 | up | 8,86 |  |
| hsa-miR-9 | 4040 | 0,27737 | 0,13 | 0,05 | -0,10 | 0,19 | -0,24 | -1,18 | dw | 12,67 |  |
| hsa-miR-297 | 6880 | 0,27851 | 0,38 | 0,08 | 0,20 | 0,13 | -0,18 | -1,13 | dw | 6,82 |  |
| hsa-miR-891a | 27961 | 0,28069 | -0,07 | 0,13 | -0,26 | 0,10 | -0,19 | -1,14 | dw | 8,06 |  |
| hsa-miR-221* | 42475 | 0,28135 | 0,18 | 0,13 | 0,02 | 0,04 | -0,16 | -1,12 | dw | 7,60 |  |
| hsa-miR-146b-3p | 42461 | 0,28793 | 0,05 | 0,21 | -0,23 | 0,11 | -0,28 | -1,21 | dw | 9,25 |  |
| hsa-let-7b* | 42769 | 0,29266 | -0,02 | 0,13 | 0,19 | 0,12 | 0,21 | 1,15 | up | 7,61 |  |
| hsa-miR-219-2-3p | 42834 | 0,29468 | -0,40 | 0,26 | 0,15 | 0,41 | 0,55 | 1,47 | up | 9,24 |  |
| hsa-miR-1 | 42573 | 0,29470 | 0,37 | 0,13 | 0,01 | 0,29 | -0,37 | -1,29 | dw | 9,34 |  |
|  |  |  |  |  |  |  |  |  |  |  |  |
| Annotation | Column ID | T-test | Mean log | Standard error | Mean log ratio | Standard error | Mean Log ratio | Foldchange | Variation | A Mean |  |
|  | Probe Id |  | Control |  | Suicide |  |  | Suicide vs Contrôle |  |  |  |
| hsa-miR-20b* | 17882 | 0,29752 | -0,02 | 0,09 | -0,14 | 0,05 | -0,11 | -1,08 | dw | 6,70 |  |
| hsa-miR-204 | 11005 | 0,30625 | 0,44 | 0,21 | 0,08 | 0,24 | -0,35 | -1,28 | dw | 8,36 |  |
| hsa-miR-196a* | 42538 | 0,30887 | -0,54 | 0,10 | -0,33 | 0,16 | 0,21 | 1,16 | up | 7,45 |  |
| hsa-miR-93 | 30687 | 0,31078 | 0,03 | 0,14 | 0,19 | 0,06 | 0,17 | 1,12 | up | 8,83 |  |
| hsa-miR-130b | 10936 | 0,31262 | 0,02 | 0,06 | 0,11 | 0,05 | 0,09 | 1,06 | up | 7,63 |  |
| hsa-miR-874 | 42808 | 0,31353 | -0,05 | 0,04 | 0,00 | 0,02 | 0,05 | 1,04 | up | 7,77 |  |
| hsa-miR-193a-3p | 10986 | 0,31602 | -0,07 | 0,22 | 0,18 | 0,07 | 0,25 | 1,19 | up | 7,62 |  |
| hsa-let-7e | 42459 | 0,32469 | 0,21 | 0,04 | 0,07 | 0,12 | -0,14 | -1,10 | dw | 13,09 |  |
| hsa-miR-498 | 42442 | 0,33155 | -0,15 | 0,11 | 0,03 | 0,13 | 0,18 | 1,13 | up | 8,44 |  |
| hsa-miR-335 | 11065 | 0,33550 | 0,38 | 0,12 | -0,04 | 0,38 | -0,42 | -1,34 | dw | 11,11 |  |
| hsa-miR-151-3p | 17463 | 0,34621 | -0,09 | 0,21 | 0,20 | 0,20 | 0,30 | 1,23 | up | 8,80 |  |
| hsa-miR-27b | 13175 | 0,35043 | -0,06 | 0,17 | 0,12 | 0,06 | 0,18 | 1,13 | up | 11,18 |  |
| hsa-miR-383 | 11098 | 0,35211 | 0,12 | 0,10 | 0,29 | 0,14 | 0,17 | 1,13 | up | 7,41 |  |
| hsa-miR-212 | 42627 | 0,35634 | 0,04 | 0,09 | -0,16 | 0,18 | -0,20 | -1,15 | dw | 8,14 |  |
| hsa-miR-95 | 11181 | 0,36160 | 0,38 | 0,14 | -0,05 | 0,42 | -0,43 | -1,35 | dw | 9,04 |  |
| hsa-miR-877 | 30033 | 0,36704 | 0,07 | 0,05 | 0,18 | 0,10 | 0,11 | 1,08 | up | 7,84 |  |
| hsa-miR-584 | 42652 | 0,37149 | -0,18 | 0,20 | 0,22 | 0,36 | 0,40 | 1,32 | up | 7,66 |  |
| hsa-miR-371-5p | 42735 | 0,37515 | -0,48 | 0,21 | -0,18 | 0,24 | 0,31 | 1,24 | up | 12,76 |  |
| hsa-miR-509-3-5p | 42589 | 0,37815 | -0,21 | 0,15 | 0,05 | 0,23 | 0,26 | 1,20 | up | 6,63 |  |
| hsa-miR-576-3p | 31057 | 0,37827 | 0,04 | 0,18 | -0,20 | 0,16 | -0,23 | -1,18 | dw | 10,21 |  |
| hsa-miR-623 | 42593 | 0,38749 | 0,08 | 0,12 | 0,24 | 0,12 | 0,15 | 1,11 | up | 6,84 |  |
| hsa-miR-487b | 14285 | 0,38775 | 0,16 | 0,12 | -0,13 | 0,29 | -0,29 | -1,22 | dw | 10,00 |  |
| hsa-miR-100 | 19581 | 0,38991 | -0,17 | 0,17 | -0,01 | 0,03 | 0,16 | 1,12 | up | 11,57 |  |
| hsa-miR-126* | 33596 | 0,40418 | 0,39 | 0,12 | -0,01 | 0,43 | -0,40 | -1,32 | dw | 8,49 |  |
| hsa-miR-923 | 42554 | 0,40834 | -0,43 | 0,36 | 0,11 | 0,49 | 0,54 | 1,45 | up | 12,62 |  |
| hsa-miR-183* | 17953 | 0,41489 | -0,13 | 0,08 | -0,01 | 0,11 | 0,12 | 1,09 | up | 7,42 |  |
| hsa-miR-582-5p | 42544 | 0,41731 | 0,32 | 0,12 | 0,03 | 0,31 | -0,29 | -1,22 | dw | 7,67 |  |
| hsa-miR-20b | 42640 | 0,42191 | 0,00 | 0,10 | -0,15 | 0,14 | -0,15 | -1,11 | dw | 7,19 |  |
| hsa-miR-137 | 10944 | 0,42404 | -0,11 | 0,08 | -0,46 | 0,40 | -0,35 | -1,28 | dw | 8,52 |  |
| hsa-miR-938 | 42776 | 0,42531 | -0,22 | 0,10 | -0,36 | 0,14 | -0,14 | -1,11 | dw | 7,16 |  |
| hsa-miR-21 | 5740 | 0,42531 | -0,02 | 0,16 | 0,15 | 0,11 | 0,17 | 1,12 | up | 9,18 |  |
| hsa-miR-495 | 42676 | 0,42630 | 0,18 | 0,13 | -0,10 | 0,30 | -0,28 | -1,21 | dw | 8,29 |  |
| hsa-miR-421 | 17474 | 0,42645 | 0,19 | 0,10 | 0,05 | 0,13 | -0,14 | -1,10 | dw | 7,23 |  |
| hsa-miR-184 | 10978 | 0,42828 | -0,01 | 0,08 | 0,13 | 0,14 | 0,13 | 1,10 | up | 8,80 |  |
|  |  |  |  |  |  |  |  |  |  |  |  |
| Annotation | Column ID | T-test | Mean log | Standard error | Mean log ratio | Standard error | Mean Log ratio | Foldchange | Variation | A Mean |  |
|  | Probe Id |  | Control |  | Suicide |  |  | Suicide vs Contrôle |  |  |  |
| hsa-miR-509-5p | 42972 | 0,42936 | -0,07 | 0,19 | -0,25 | 0,10 | -0,18 | -1,13 | dw | 8,97 |  |
| hsa-miR-539 | 14271 | 0,43012 | 0,36 | 0,08 | 0,24 | 0,11 | -0,12 | -1,08 | dw | 6,83 |  |
| hsa-miR-7 | 3980 | 0,43316 | 0,12 | 0,09 | -0,10 | 0,25 | -0,22 | -1,17 | dw | 11,71 |  |
| hsa-let-7f | 17752 | 0,43443 | 0,17 | 0,16 | -0,18 | 0,39 | -0,35 | -1,27 | dw | 8,05 |  |
| hsa-miR-125b | 30787 | 0,43816 | -0,26 | 0,06 | -0,18 | 0,07 | 0,08 | 1,06 | up | 15,11 |  |
| hsa-miR-451 | 42866 | 0,43855 | 0,06 | 0,39 | -0,31 | 0,20 | -0,36 | -1,29 | dw | 12,70 |  |
| hsa-miR-30e* | 11224 | 0,44536 | 0,31 | 0,15 | 0,10 | 0,21 | -0,21 | -1,16 | dw | 8,99 |  |
| hsa-miR-423-3p | 42730 | 0,45127 | -0,03 | 0,12 | 0,10 | 0,11 | 0,13 | 1,10 | up | 8,91 |  |
| hsa-miR-518b | 42643 | 0,45553 | -0,03 | 0,24 | 0,18 | 0,08 | 0,20 | 1,15 | up | 8,36 |  |
| hsa-miR-32 | 11053 | 0,46215 | -0,04 | 0,22 | 0,14 | 0,07 | 0,18 | 1,13 | up | 7,29 |  |
| hsa-miR-126 | 4610 | 0,46318 | 0,19 | 0,13 | -0,11 | 0,36 | -0,30 | -1,23 | dw | 12,40 |  |
| hsa-miR-323-3p | 42957 | 0,46384 | 0,06 | 0,10 | -0,18 | 0,28 | -0,23 | -1,17 | dw | 7,54 |  |
| hsa-miR-622 | 17493 | 0,46872 | 0,08 | 0,08 | 0,23 | 0,19 | 0,16 | 1,12 | up | 6,93 |  |
| hsa-miR-340* | 42738 | 0,47058 | 0,21 | 0,06 | 0,06 | 0,18 | -0,15 | -1,11 | dw | 7,60 |  |
| hsa-miR-340 | 29872 | 0,47113 | -0,03 | 0,10 | 0,08 | 0,10 | 0,10 | 1,08 | up | 10,30 |  |
| hsa-miR-25* | 42929 | 0,47716 | -0,22 | 0,07 | -0,13 | 0,09 | 0,09 | 1,06 | up | 7,90 |  |
| hsa-miR-181b | 10972 | 0,47818 | -0,04 | 0,18 | 0,14 | 0,14 | 0,18 | 1,13 | up | 9,07 |  |
| hsa-miR-409-5p | 42448 | 0,48067 | -0,44 | 0,10 | -0,35 | 0,06 | 0,09 | 1,06 | up | 7,29 |  |
| hsa-miR-519c-5p/hsa-miR-519b-5p/hsa-miR-523*/hsa-miR-518e* | 13137 | 0,48120 | -0,12 | 0,26 | -0,37 | 0,19 | -0,24 | -1,18 | dw | 9,66 |  |
| hsa-miR-129-3p | 42583 | 0,48350 | 0,17 | 0,14 | -0,07 | 0,29 | -0,24 | -1,18 | dw | 12,34 |  |
| hsa-miR-708 | 29190 | 0,48453 | 0,22 | 0,08 | -0,03 | 0,32 | -0,25 | -1,19 | dw | 9,82 |  |
| hsa-miR-876-3p | 28884 | 0,48494 | 0,17 | 0,04 | 0,03 | 0,19 | -0,14 | -1,10 | dw | 6,95 |  |
| hsa-miR-135a | 42839 | 0,48662 | 0,26 | 0,22 | -0,06 | 0,37 | -0,32 | -1,25 | dw | 10,50 |  |
| hsa-miR-186 | 18739 | 0,48873 | 0,21 | 0,12 | 0,30 | 0,03 | 0,09 | 1,07 | up | 7,73 |  |
| hsa-miR-494 | 42540 | 0,49017 | -0,34 | 0,04 | -0,41 | 0,08 | -0,07 | -1,05 | dw | 10,61 |  |
| hsa-miR-519e* | 13132 | 0,49104 | -0,36 | 0,09 | -0,25 | 0,13 | 0,11 | 1,08 | up | 8,82 |  |
| hsa-miR-374b* | 42476 | 0,49948 | 0,08 | 0,11 | -0,01 | 0,07 | -0,09 | -1,07 | dw | 8,76 |  |
| hsa-miR-199a-5p | 29562 | 0,50058 | 0,29 | 0,15 | 0,15 | 0,12 | -0,14 | -1,10 | dw | 7,03 |  |
| hsa-miR-934 | 17863 | 0,50128 | -0,14 | 0,19 | -0,30 | 0,10 | -0,15 | -1,11 | dw | 8,95 |  |
| hsa-miR-101 | 31026 | 0,50183 | -0,14 | 0,17 | -0,01 | 0,05 | 0,13 | 1,09 | up | 11,46 |  |
| hsa-miR-635 | 17391 | 0,51020 | -0,26 | 0,16 | -0,43 | 0,18 | -0,17 | -1,12 | dw | 7,52 |  |
| hsa-miR-376a* | 42885 | 0,51047 | 0,26 | 0,10 | 0,06 | 0,27 | -0,20 | -1,15 | dw | 8,87 |  |
| hsa-miR-411 | 17482 | 0,51276 | 0,30 | 0,13 | 0,03 | 0,36 | -0,27 | -1,21 | dw | 8,74 |  |
| hsa-miR-99b | 11184 | 0,51285 | -0,06 | 0,13 | 0,05 | 0,10 | 0,11 | 1,08 | up | 10,92 |  |
| Annotation | Column ID | T-test | Mean log | Standard error | Mean log ratio | Standard error | Mean Log ratio | Foldchange | Variation | A Mean |  |
|  | Probe Id |  | Control |  | Suicide |  |  | Suicide vs Contrôle |  |  |  |
| hsa-miR-153 | 42599 | 0,51998 | 0,38 | 0,16 | 0,05 | 0,45 | -0,33 | -1,25 | dw | 9,78 |  |
| hsa-miR-139-5p | 27542 | 0,52088 | 0,25 | 0,13 | 0,03 | 0,29 | -0,21 | -1,16 | dw | 7,99 |  |
| hsa-miR-298 | 42671 | 0,52228 | -0,24 | 0,13 | -0,01 | 0,31 | 0,23 | 1,17 | up | 9,51 |  |
| hsa-miR-143 | 13177 | 0,52352 | 0,26 | 0,17 | 0,09 | 0,20 | -0,17 | -1,13 | dw | 11,50 |  |
| hsa-miR-20a | 42649 | 0,52421 | 0,21 | 0,15 | 0,10 | 0,06 | -0,11 | -1,08 | dw | 10,40 |  |
| hsa-miR-152 | 17676 | 0,52652 | 0,14 | 0,18 | 0,27 | 0,06 | 0,13 | 1,09 | up | 7,25 |  |
| hsa-miR-33a | 42716 | 0,52660 | -0,12 | 0,25 | 0,06 | 0,06 | 0,17 | 1,13 | up | 8,99 |  |
| hsa-miR-24 | 17506 | 0,53344 | 0,07 | 0,11 | 0,15 | 0,04 | 0,08 | 1,06 | up | 12,34 |  |
| hsa-miR-411* | 42784 | 0,53580 | 0,17 | 0,11 | -0,02 | 0,28 | -0,20 | -1,15 | dw | 7,67 |  |
| miRPlus_42521 | 42521 | 0,53789 | -0,19 | 0,27 | 0,02 | 0,16 | 0,21 | 1,15 | up | 10,76 |  |
| hsa-miR-504 | 28480 | 0,53798 | 0,18 | 0,10 | 0,02 | 0,21 | -0,15 | -1,11 | dw | 7,18 |  |
| hsa-miR-187* | 42775 | 0,53841 | -0,44 | 0,11 | -0,29 | 0,19 | 0,15 | 1,11 | up | 8,95 |  |
| hsa-miR-30b | 17565 | 0,54181 | -0,13 | 0,09 | -0,28 | 0,22 | -0,16 | -1,11 | dw | 11,72 |  |
| hsa-miR-129* | 42571 | 0,54399 | 0,17 | 0,14 | -0,04 | 0,29 | -0,21 | -1,16 | dw | 12,53 |  |
| hsa-miR-99a | 42708 | 0,54414 | 0,08 | 0,16 | -0,10 | 0,23 | -0,18 | -1,13 | dw | 9,81 |  |
| hsa-miR-214 | 11014 | 0,54666 | -0,05 | 0,12 | -0,13 | 0,06 | -0,08 | -1,06 | dw | 8,46 |  |
| hsa-miR-503 | 11135 | 0,54950 | -0,07 | 0,35 | -0,32 | 0,19 | -0,25 | -1,19 | dw | 11,97 |  |
| hsa-miR-22* | 42532 | 0,55982 | 0,20 | 0,12 | 0,10 | 0,13 | -0,11 | -1,08 | dw | 8,40 |  |
| hsa-miR-300 | 42513 | 0,56278 | -0,08 | 0,20 | 0,05 | 0,08 | 0,13 | 1,09 | up | 10,76 |  |
| hsa-miR-26b | 42564 | 0,56560 | 0,08 | 0,06 | 0,00 | 0,13 | -0,09 | -1,06 | dw | 13,69 |  |
| hsa-let-7a | 17748 | 0,56790 | 0,10 | 0,10 | -0,02 | 0,18 | -0,12 | -1,09 | dw | 13,35 |  |
| hsa-miR-124* | 42898 | 0,56807 | 0,04 | 0,02 | -0,05 | 0,15 | -0,09 | -1,06 | dw | 7,64 |  |
| hsa-miR-379 | 11093 | 0,56825 | 0,32 | 0,14 | 0,08 | 0,37 | -0,24 | -1,18 | dw | 9,34 |  |
| hsa-miR-487a | 13183 | 0,57053 | 0,20 | 0,10 | 0,04 | 0,25 | -0,16 | -1,12 | dw | 8,17 |  |
| hsa-miR-331-3p | 42887 | 0,57184 | 0,12 | 0,13 | -0,06 | 0,28 | -0,18 | -1,14 | dw | 10,04 |  |
| hsa-miR-9* | 29852 | 0,57597 | 0,17 | 0,04 | 0,04 | 0,21 | -0,13 | -1,09 | dw | 13,70 |  |
| hsa-miR-181a* | 11013 | 0,57691 | -0,07 | 0,13 | 0,01 | 0,04 | 0,08 | 1,06 | up | 7,47 |  |
| hsa-miR-142-3p | 10947 | 0,57891 | 0,21 | 0,18 | 0,09 | 0,08 | -0,12 | -1,08 | dw | 8,52 |  |
| hsa-miR-379* | 42552 | 0,58130 | 0,06 | 0,13 | -0,11 | 0,27 | -0,17 | -1,13 | dw | 7,19 |  |
| hsa-miR-195 | 13148 | 0,58142 | 0,03 | 0,13 | -0,16 | 0,31 | -0,20 | -1,15 | dw | 11,11 |  |
| hsa-miR-151-5p | 11260 | 0,58221 | -0,01 | 0,18 | 0,10 | 0,04 | 0,10 | 1,08 | up | 10,02 |  |
| hsa-miR-130a | 10138 | 0,58258 | 0,15 | 0,13 | -0,03 | 0,28 | -0,18 | -1,13 | dw | 10,30 |  |
| hsa-miR-222 | 11023 | 0,58277 | 0,10 | 0,09 | -0,03 | 0,19 | -0,12 | -1,09 | dw | 11,71 |  |
| hsa-miR-197 | 42783 | 0,58436 | -0,15 | 0,03 | -0,20 | 0,07 | -0,05 | -1,03 | dw | 8,62 |  |
|  |  |  |  |  |  |  |  |  |  |  |  |
| Annotation | Column ID | T-test | Mean log | Standard error | Mean log ratio | Standard error | Mean Log ratio | Foldchange | Variation | A Mean |  |
|  | Probe Id |  | Control |  | Suicide |  |  | Suicide vs Contrôle |  |  |  |
| hsa-miR-659 | 42749 | 0,58546 | -0,20 | 0,10 | 0,00 | 0,33 | 0,20 | 1,15 | up | 8,23 |  |
| hsa-miR-363 | 11077 | 0,59069 | 0,20 | 0,12 | 0,07 | 0,20 | -0,13 | -1,10 | dw | 8,07 |  |
| hsa-miR-526b | 11176 | 0,59213 | -0,16 | 0,12 | 0,02 | 0,30 | 0,19 | 1,14 | up | 8,55 |  |
| hsa-miR-625 | 17573 | 0,59221 | -0,01 | 0,07 | 0,05 | 0,10 | 0,07 | 1,05 | up | 9,20 |  |
| hsa-miR-190 | 27536 | 0,60045 | 0,43 | 0,14 | 0,25 | 0,30 | -0,18 | -1,14 | dw | 8,36 |  |
| hsa-miR-99a* | 17852 | 0,60404 | 0,32 | 0,10 | 0,23 | 0,13 | -0,09 | -1,06 | dw | 6,86 |  |
| hsa-miR-574-3p | 28966 | 0,60465 | 0,00 | 0,08 | -0,07 | 0,09 | -0,07 | -1,05 | dw | 8,76 |  |
| hsa-miR-557 | 42760 | 0,60484 | -0,35 | 0,07 | -0,23 | 0,21 | 0,12 | 1,09 | up | 7,54 |  |
| hsa-miR-16 | 10967 | 0,60722 | 0,06 | 0,13 | -0,04 | 0,14 | -0,10 | -1,07 | dw | 11,92 |  |
| hsa-miR-424 | 42965 | 0,60871 | -0,09 | 0,11 | -0,16 | 0,08 | -0,07 | -1,05 | dw | 9,43 |  |
| hsa-miR-409-3p | 11240 | 0,61139 | 0,01 | 0,18 | 0,19 | 0,27 | 0,17 | 1,13 | up | 7,34 |  |
| hsa-miR-939 | 42529 | 0,61230 | -0,03 | 0,19 | 0,12 | 0,20 | 0,15 | 1,11 | up | 11,09 |  |
| hsa-miR-374b | 14302 | 0,61539 | -0,13 | 0,15 | -0,04 | 0,09 | 0,09 | 1,07 | up | 8,48 |  |
| hsa-miR-433 | 42853 | 0,61772 | 0,09 | 0,12 | 0,18 | 0,12 | 0,09 | 1,06 | up | 7,71 |  |
| hsa-miR-193b* | 42962 | 0,61878 | -0,07 | 0,15 | 0,03 | 0,14 | 0,11 | 1,08 | up | 7,58 |  |
| hsa-miR-154* | 42572 | 0,61946 | 0,15 | 0,10 | -0,01 | 0,29 | -0,16 | -1,12 | dw | 7,32 |  |
| hsa-miR-218 | 11018 | 0,62078 | 0,06 | 0,11 | -0,06 | 0,21 | -0,12 | -1,09 | dw | 8,75 |  |
| hsa-miR-27a | 19593 | 0,62461 | -0,02 | 0,14 | 0,07 | 0,10 | 0,09 | 1,06 | up | 10,18 |  |
| hsa-miR-208a | 5730 | 0,62544 | -0,23 | 0,15 | -0,15 | 0,04 | 0,08 | 1,06 | up | 7,26 |  |
| hsa-miR-675 | 42761 | 0,62745 | -0,37 | 0,19 | -0,20 | 0,27 | 0,17 | 1,12 | up | 7,86 |  |
| hsa-miR-615-3p | 27672 | 0,63156 | 0,06 | 0,07 | 0,01 | 0,08 | -0,06 | -1,04 | dw | 7,50 |  |
| hsa-miR-765 | 28150 | 0,63455 | 0,09 | 0,23 | -0,07 | 0,24 | -0,17 | -1,12 | dw | 11,19 |  |
| hsa-miR-185 | 5560 | 0,63486 | -0,07 | 0,06 | 0,00 | 0,13 | 0,07 | 1,05 | up | 11,29 |  |
| hsa-miR-23a | 42744 | 0,63867 | -0,07 | 0,14 | 0,03 | 0,14 | 0,10 | 1,07 | up | 11,11 |  |
| hsa-miR-665 | 42762 | 0,64465 | -0,24 | 0,17 | -0,05 | 0,36 | 0,19 | 1,14 | up | 12,75 |  |
| hsa-miR-28-5p | 42831 | 0,65263 | -0,09 | 0,19 | 0,00 | 0,04 | 0,09 | 1,07 | up | 9,57 |  |
| hsa-miR-133a | 32809 | 0,65378 | 0,32 | 0,15 | 0,20 | 0,20 | -0,12 | -1,09 | dw | 7,48 |  |
| hsa-miR-132* | 17808 | 0,65500 | 0,21 | 0,10 | 0,09 | 0,22 | -0,11 | -1,08 | dw | 8,74 |  |
| hsa-miR-744 | 27559 | 0,65614 | 0,12 | 0,11 | -0,02 | 0,27 | -0,14 | -1,10 | dw | 10,95 |  |
| hsa-miR-29a | 11039 | 0,66238 | -0,21 | 0,12 | -0,14 | 0,08 | 0,07 | 1,05 | up | 14,01 |  |
| hsa-miR-30c-2* | 42701 | 0,66266 | 0,10 | 0,16 | -0,02 | 0,20 | -0,12 | -1,09 | dw | 10,75 |  |
| hsa-miR-132 | 10937 | 0,66560 | 0,14 | 0,13 | 0,01 | 0,25 | -0,13 | -1,09 | dw | 9,73 |  |
| hsa-miR-483-5p | 42654 | 0,66909 | -0,14 | 0,13 | -0,03 | 0,20 | 0,11 | 1,08 | up | 7,90 |  |
| hsa-miR-329 | 11061 | 0,67384 | 0,11 | 0,14 | -0,04 | 0,30 | -0,15 | -1,11 | dw | 9,03 |  |
|  |  |  |  |  |  |  |  |  |  |  |  |
| Annotation | Column ID | T-test | Mean log | Standard error | Mean log ratio | Standard error | Mean Log ratio | Foldchange | Variation | A Mean |  |
|  | Probe Id |  | Control |  | Suicide |  |  | Suicide vs Contrôle |  |  |  |
| hsa-miR-193b | 10987 | 0,67820 | 0,02 | 0,19 | 0,12 | 0,11 | 0,10 | 1,07 | up | 8,55 |  |
| hsa-miR-490-3p | 42703 | 0,68108 | -0,20 | 0,17 | -0,30 | 0,15 | -0,10 | -1,07 | dw | 8,19 |  |
| hsa-miR-34c-5p | 11074 | 0,68343 | 0,19 | 0,05 | 0,24 | 0,10 | 0,05 | 1,04 | up | 7,27 |  |
| hsa-miR-22 | 11020 | 0,68363 | 0,12 | 0,09 | 0,19 | 0,12 | 0,06 | 1,05 | up | 12,56 |  |
| hsa-miR-342-3p | 32884 | 0,69597 | 0,07 | 0,11 | -0,03 | 0,23 | -0,11 | -1,08 | dw | 10,86 |  |
| hsa-miR-147 | 10954 | 0,69889 | -0,10 | 0,09 | -0,06 | 0,05 | 0,04 | 1,03 | up | 6,97 |  |
| hsa-miR-98 | 11182 | 0,69914 | 0,20 | 0,14 | 0,08 | 0,26 | -0,12 | -1,09 | dw | 9,63 |  |
| hsa-miR-15a | 27720 | 0,69978 | 0,01 | 0,13 | 0,06 | 0,04 | 0,06 | 1,04 | up | 10,29 |  |
| hsa-miR-519e | 11164 | 0,70108 | -0,18 | 0,11 | -0,13 | 0,07 | 0,05 | 1,04 | up | 7,38 |  |
| hsa-miR-638 | 42832 | 0,70606 | -0,12 | 0,26 | 0,01 | 0,22 | 0,13 | 1,10 | up | 8,38 |  |
| hsa-miR-128 | 33902 | 0,70624 | -0,10 | 0,06 | -0,21 | 0,27 | -0,11 | -1,08 | dw | 12,03 |  |
| hsa-miR-382 | 11097 | 0,70701 | 0,05 | 0,08 | -0,01 | 0,13 | -0,06 | -1,04 | dw | 8,17 |  |
| hsa-miR-551b* | 42781 | 0,70839 | -0,27 | 0,14 | -0,13 | 0,30 | 0,13 | 1,09 | up | 8,48 |  |
| hsa-miR-140-3p | 42630 | 0,71109 | 0,00 | 0,17 | 0,07 | 0,07 | 0,07 | 1,05 | up | 10,21 |  |
| hsa-miR-30b* | 17836 | 0,71304 | -0,04 | 0,18 | 0,07 | 0,24 | 0,12 | 1,08 | up | 10,90 |  |
| miRPlus_42526 | 42526 | 0,71432 | 0,04 | 0,38 | -0,13 | 0,22 | -0,17 | -1,12 | dw | 13,76 |  |
| hsa-miR-524-5p | 10618 | 0,72546 | 0,08 | 0,08 | 0,04 | 0,09 | -0,04 | -1,03 | dw | 7,79 |  |
| hsa-miR-296-3p | 42528 | 0,72818 | 0,05 | 0,09 | 0,13 | 0,21 | 0,08 | 1,06 | up | 6,64 |  |
| hsa-miR-142-5p | 19015 | 0,72995 | 0,08 | 0,14 | 0,13 | 0,07 | 0,06 | 1,04 | up | 7,87 |  |
| hsa-miR-181a-2* | 17928 | 0,73294 | -0,31 | 0,04 | -0,27 | 0,10 | 0,04 | 1,03 | up | 6,55 |  |
| hsa-miR-146b-5p | 10306 | 0,73314 | 0,23 | 0,11 | 0,13 | 0,25 | -0,10 | -1,07 | dw | 9,87 |  |
| hsa-miR-497 | 42847 | 0,73394 | -0,15 | 0,10 | -0,23 | 0,19 | -0,08 | -1,05 | dw | 7,33 |  |
| hsa-miR-154 | 10962 | 0,73634 | 0,06 | 0,14 | -0,02 | 0,18 | -0,08 | -1,06 | dw | 9,31 |  |
| miRPlus_42745 | 42745 | 0,74058 | -0,21 | 0,30 | -0,08 | 0,25 | 0,14 | 1,10 | up | 11,03 |  |
| hsa-miR-574-5p | 27740 | 0,74706 | 0,11 | 0,18 | 0,02 | 0,21 | -0,09 | -1,07 | dw | 10,13 |  |
| hsa-miR-617 | 17552 | 0,75453 | 0,10 | 0,14 | 0,05 | 0,08 | -0,05 | -1,04 | dw | 8,05 |  |
| hsa-miR-198 | 27540 | 0,75474 | 0,10 | 0,18 | 0,00 | 0,23 | -0,10 | -1,07 | dw | 7,83 |  |
| hsa-miR-886-3p | 42806 | 0,75676 | 0,00 | 0,26 | -0,09 | 0,12 | -0,09 | -1,07 | dw | 8,77 |  |
| hsa-miR-500* | 42600 | 0,75912 | 0,11 | 0,09 | 0,07 | 0,08 | -0,04 | -1,03 | dw | 7,28 |  |
| hsa-miR-365 | 11078 | 0,76053 | 0,17 | 0,11 | 0,09 | 0,24 | -0,08 | -1,06 | dw | 9,15 |  |
| miRPlus_17955 | 17955 | 0,76059 | -0,21 | 0,04 | -0,29 | 0,23 | -0,08 | -1,05 | dw | 8,00 |  |
| hsa-miR-302d* | 42820 | 0,76063 | -0,12 | 0,16 | -0,04 | 0,17 | 0,07 | 1,05 | up | 11,02 |  |
| hsa-miR-30c | 42923 | 0,76095 | -0,01 | 0,09 | -0,07 | 0,15 | -0,06 | -1,04 | dw | 12,66 |  |
| hsa-miR-553 | 29460 | 0,77032 | 0,00 | 0,13 | -0,07 | 0,17 | -0,06 | -1,05 | dw | 6,98 |  |
|  |  |  |  |  |  |  |  |  |  |  |  |
| Annotation | Column ID | T-test | Mean log | Standard error | Mean log ratio | Standard error | Mean Log ratio | Foldchange | Variation | A Mean |  |
|  | Probe Id |  | Control |  | Suicide |  |  | Suicide vs Contrôle |  |  |  |
| hsa-miR-516b | 11151 | 0,77174 | -0,07 | 0,17 | -0,14 | 0,14 | -0,07 | -1,05 | dw | 7,74 |  |
| hsa-miR-337-5p | 17944 | 0,77186 | 0,18 | 0,14 | 0,12 | 0,16 | -0,06 | -1,05 | dw | 7,00 |  |
| hsa-miR-29a* | 42920 | 0,77372 | 0,09 | 0,18 | 0,15 | 0,10 | 0,06 | 1,04 | up | 7,30 |  |
| hsa-miR-583 | 17295 | 0,77670 | -0,05 | 0,21 | -0,14 | 0,23 | -0,09 | -1,07 | dw | 10,49 |  |
| hsa-miR-628-3p | 42958 | 0,77679 | -0,07 | 0,27 | 0,02 | 0,15 | 0,09 | 1,06 | up | 11,83 |  |
| hsa-miR-223 | 11024 | 0,77721 | 0,08 | 0,15 | 0,13 | 0,08 | 0,05 | 1,04 | up | 8,45 |  |
| hsa-miR-488 | 42704 | 0,77747 | 0,11 | 0,13 | 0,07 | 0,06 | -0,04 | -1,03 | dw | 9,90 |  |
| hsa-miR-485-5p | 42480 | 0,77818 | 0,26 | 0,11 | 0,20 | 0,17 | -0,06 | -1,04 | dw | 6,99 |  |
| hsa-miR-31 | 11052 | 0,77962 | 0,11 | 0,19 | 0,19 | 0,19 | 0,08 | 1,06 | up | 7,60 |  |
| hsa-let-7d* | 42505 | 0,78070 | 0,09 | 0,07 | 0,11 | 0,02 | 0,02 | 1,01 | up | 7,37 |  |
| hsa-miR-422a | 11104 | 0,78074 | 0,11 | 0,23 | 0,00 | 0,27 | -0,10 | -1,08 | dw | 8,80 |  |
| hsa-miR-221 | 11022 | 0,78559 | 0,06 | 0,13 | -0,01 | 0,20 | -0,07 | -1,05 | dw | 9,70 |  |
| hsa-miR-549 | 42798 | 0,78746 | -0,05 | 0,14 | -0,09 | 0,10 | -0,05 | -1,03 | dw | 9,58 |  |
| hsa-miR-125a-3p | 17942 | 0,78800 | 0,44 | 0,08 | 0,46 | 0,05 | 0,03 | 1,02 | up | 6,59 |  |
| hsa-miR-136* | 42512 | 0,79418 | 0,00 | 0,14 | -0,07 | 0,20 | -0,07 | -1,05 | dw | 8,72 |  |
| hsa-miR-518c* | 13131 | 0,79700 | -0,18 | 0,17 | -0,09 | 0,28 | 0,09 | 1,06 | up | 11,31 |  |
| hsa-miR-657 | 17460 | 0,80036 | -0,10 | 0,18 | -0,05 | 0,07 | 0,05 | 1,04 | up | 6,59 |  |
| hsa-miR-516a-5p | 42550 | 0,80076 | -0,12 | 0,12 | -0,18 | 0,17 | -0,06 | -1,04 | dw | 8,56 |  |
| hsa-let-7g | 19602 | 0,80189 | 0,01 | 0,14 | -0,06 | 0,20 | -0,06 | -1,05 | dw | 7,57 |  |
| hsa-miR-620 | 42994 | 0,81063 | -0,08 | 0,21 | -0,16 | 0,20 | -0,07 | -1,05 | dw | 12,08 |  |
| hsa-miR-136 | 10943 | 0,81084 | -0,14 | 0,25 | -0,07 | 0,05 | 0,06 | 1,04 | up | 9,11 |  |
| hsa-miR-600 | 17377 | 0,81147 | -0,07 | 0,07 | -0,04 | 0,08 | 0,03 | 1,02 | up | 7,90 |  |
| hsa-miR-299-5p | 11038 | 0,81306 | 0,49 | 0,14 | 0,43 | 0,18 | -0,06 | -1,04 | dw | 7,37 |  |
| hsa-miR-136* | 42686 | 0,81423 | -0,14 | 0,17 | -0,09 | 0,14 | 0,05 | 1,04 | up | 7,95 |  |
| hsa-miR-885-5p | 17830 | 0,82074 | -0,06 | 0,04 | -0,03 | 0,12 | 0,03 | 1,02 | up | 10,39 |  |
| hsa-miR-768-3p | 27898 | 0,82248 | -0,02 | 0,15 | -0,08 | 0,16 | -0,05 | -1,04 | dw | 11,53 |  |
| hsa-miR-377 | 11091 | 0,82324 | 0,07 | 0,15 | 0,00 | 0,26 | -0,07 | -1,05 | dw | 9,89 |  |
| hsa-miR-518a-5p/hsa-miR-527 | 11177 | 0,82434 | -0,19 | 0,07 | -0,15 | 0,20 | 0,05 | 1,04 | up | 9,91 |  |
| hsa-miR-34a | 27217 | 0,82506 | 0,05 | 0,21 | -0,02 | 0,24 | -0,07 | -1,05 | dw | 10,26 |  |
| hsa-miR-106a | 42648 | 0,83091 | 0,09 | 0,15 | 0,05 | 0,05 | -0,03 | -1,02 | dw | 9,38 |  |
| hsa-miR-500 | 17875 | 0,83302 | 0,11 | 0,05 | 0,09 | 0,09 | -0,02 | -1,02 | dw | 6,81 |  |
| hsa-miR-181c | 42496 | 0,83940 | -0,13 | 0,12 | -0,19 | 0,28 | -0,06 | -1,05 | dw | 6,87 |  |
| hsa-miR-17 | 42650 | 0,83960 | 0,08 | 0,15 | 0,11 | 0,04 | 0,03 | 1,02 | up | 9,46 |  |
| hsa-miR-376c | 42629 | 0,84089 | -0,01 | 0,12 | -0,07 | 0,25 | -0,06 | -1,04 | dw | 9,29 |  |
|  |  |  |  |  |  |  |  |  |  |  |  |
| Annotation | Column ID | T-test | Mean log | Standard error | Mean log ratio | Standard error | Mean Log ratio | Foldchange | Variation | A Mean |  |
|  | Probe Id |  | Control |  | Suicide |  |  | Suicide vs Contrôle |  |  |  |
| hsa-miR-876-5p | 42940 | 0,84224 | 0,08 | 0,13 | 0,04 | 0,12 | -0,04 | -1,03 | dw | 7,75 |  |
| hsa-miR-378 | 11105 | 0,84450 | 0,09 | 0,10 | 0,06 | 0,12 | -0,03 | -1,02 | dw | 8,14 |  |
| hsa-miR-640 | 42758 | 0,84461 | -0,41 | 0,22 | -0,47 | 0,24 | -0,07 | -1,05 | dw | 6,58 |  |
| hsa-miR-933 | 42539 | 0,84906 | -0,27 | 0,02 | -0,31 | 0,17 | -0,03 | -1,02 | dw | 12,69 |  |
| hsa-miR-374a | 27378 | 0,84955 | 0,21 | 0,12 | 0,18 | 0,14 | -0,04 | -1,03 | dw | 10,37 |  |
| hsa-miR-124 | 14328 | 0,85055 | 0,21 | 0,07 | 0,24 | 0,10 | 0,02 | 1,02 | up | 15,37 |  |
| hsa-miR-769-5p | 32891 | 0,85065 | 0,12 | 0,11 | 0,08 | 0,17 | -0,04 | -1,03 | dw | 9,40 |  |
| hsa-miR-138 | 13140 | 0,85162 | 0,07 | 0,09 | 0,11 | 0,23 | 0,05 | 1,03 | up | 12,27 |  |
| hsa-miR-195* | 42723 | 0,85483 | -0,03 | 0,20 | -0,08 | 0,22 | -0,06 | -1,04 | dw | 7,75 |  |
| hsa-miR-320a | 27533 | 0,85709 | 0,09 | 0,14 | 0,11 | 0,04 | 0,03 | 1,02 | up | 10,46 |  |
| hsa-miR-133b | 30755 | 0,85921 | 0,25 | 0,13 | 0,20 | 0,19 | -0,04 | -1,03 | dw | 7,87 |  |
| hsa-miR-342-5p | 42576 | 0,86759 | -0,12 | 0,08 | -0,09 | 0,13 | 0,03 | 1,02 | up | 6,86 |  |
| hsa-miR-598 | 17637 | 0,86977 | 0,02 | 0,14 | -0,02 | 0,21 | -0,04 | -1,03 | dw | 7,99 |  |
| hsa-miR-24-1* | 42575 | 0,87225 | 0,10 | 0,07 | 0,08 | 0,05 | -0,01 | -1,01 | dw | 8,51 |  |
| hsa-miR-29c | 11041 | 0,87306 | -0,26 | 0,17 | -0,23 | 0,12 | 0,04 | 1,02 | up | 11,70 |  |
| hsa-miR-30e | 28191 | 0,87338 | 0,21 | 0,17 | 0,16 | 0,23 | -0,05 | -1,03 | dw | 10,24 |  |
| hsa-miR-520d-5p | 42537 | 0,87453 | -0,38 | 0,06 | -0,40 | 0,07 | -0,02 | -1,01 | dw | 11,66 |  |
| hsa-miR-191 | 10985 | 0,87552 | 0,01 | 0,12 | 0,04 | 0,15 | 0,03 | 1,02 | up | 11,24 |  |
| miRPlus_17848 | 17848 | 0,87617 | -0,10 | 0,21 | -0,07 | 0,05 | 0,03 | 1,02 | up | 6,86 |  |
| hsa-let-7i | 19580 | 0,87661 | 0,08 | 0,11 | 0,11 | 0,12 | 0,03 | 1,02 | up | 11,18 |  |
| hsa-miR-425 | 17608 | 0,87675 | -0,01 | 0,13 | 0,03 | 0,17 | 0,04 | 1,02 | up | 8,16 |  |
| hsa-miR-920 | 42932 | 0,87788 | -0,10 | 0,11 | -0,13 | 0,15 | -0,03 | -1,02 | dw | 8,21 |  |
| hsa-miR-324-5p | 42477 | 0,87814 | 0,06 | 0,11 | 0,10 | 0,19 | 0,04 | 1,02 | up | 7,70 |  |
| hsa-miR-658 | 17522 | 0,87844 | -0,12 | 0,25 | -0,17 | 0,25 | -0,06 | -1,04 | dw | 11,85 |  |
| hsa-miR-376a | 11089 | 0,88501 | -0,05 | 0,12 | -0,08 | 0,15 | -0,03 | -1,02 | dw | 7,96 |  |
| hsa-miR-200b* | 17859 | 0,88730 | -0,10 | 0,17 | -0,07 | 0,11 | 0,03 | 1,02 | up | 8,27 |  |
| hsa-miR-629* | 17566 | 0,88907 | -0,03 | 0,03 | -0,04 | 0,02 | 0,00 | -1,00 | dw | 6,72 |  |
| miRPlus_42793 | 42793 | 0,89234 | -0,24 | 0,15 | -0,19 | 0,25 | 0,04 | 1,03 | up | 9,76 |  |
| hsa-miR-140-5p | 4700 | 0,89239 | 0,17 | 0,14 | 0,15 | 0,12 | -0,03 | -1,02 | dw | 8,34 |  |
| hsa-miR-491-5p | 11123 | 0,89793 | 0,08 | 0,18 | 0,04 | 0,22 | -0,04 | -1,03 | dw | 8,40 |  |
| hsa-miR-505* | 42490 | 0,89907 | 0,10 | 0,14 | 0,13 | 0,21 | 0,03 | 1,02 | up | 8,25 |  |
| hsa-miR-146a | 10952 | 0,91025 | 0,18 | 0,09 | 0,16 | 0,14 | -0,02 | -1,01 | dw | 7,40 |  |
| hsa-miR-23b | 11027 | 0,91362 | 0,03 | 0,07 | 0,04 | 0,07 | 0,01 | 1,01 | up | 12,72 |  |
| hsa-let-7e* | 42743 | 0,91408 | 0,28 | 0,09 | 0,26 | 0,13 | -0,02 | -1,01 | dw | 6,56 |  |
|  |  |  |  |  |  |  |  |  |  |  |  |
| Annotation | Column ID | T-test | Mean log | Standard error | Mean log ratio | Standard error | Mean Log ratio | Foldchange | Variation | A Mean |  |
|  | Probe Id |  | Control |  | Suicide |  |  | Suicide vs Contrôle |  |  |  |
| hsa-miR-103 | 10919 | 0,91515 | 0,04 | 0,08 | 0,02 | 0,17 | -0,02 | -1,01 | dw | 11,13 |  |
| hsa-miR-149 | 42810 | 0,91525 | 0,00 | 0,16 | -0,03 | 0,25 | -0,03 | -1,02 | dw | 9,24 |  |
| hsa-miR-15b | 17280 | 0,91995 | 0,06 | 0,15 | 0,07 | 0,06 | 0,02 | 1,01 | up | 8,97 |  |
| hsa-miR-514 | 42959 | 0,92018 | -0,05 | 0,17 | -0,03 | 0,10 | 0,02 | 1,01 | up | 7,43 |  |
| hsa-miR-510 | 11142 | 0,92395 | -0,22 | 0,14 | -0,20 | 0,20 | 0,02 | 1,02 | up | 8,44 |  |
| hsa-miR-29c* | 14300 | 0,92964 | -0,03 | 0,19 | -0,01 | 0,18 | 0,02 | 1,02 | up | 8,06 |  |
| hsa-miR-326 | 42693 | 0,93014 | 0,19 | 0,06 | 0,19 | 0,05 | 0,01 | 1,00 | up | 7,46 |  |
| hsa-miR-301a | 13143 | 0,93257 | 0,17 | 0,12 | 0,19 | 0,21 | 0,02 | 1,01 | up | 9,44 |  |
| hsa-miR-127-5p | 42692 | 0,94307 | 0,02 | 0,14 | 0,04 | 0,17 | 0,02 | 1,01 | up | 8,08 |  |
| hsa-miR-150 | 42802 | 0,94327 | -0,09 | 0,13 | -0,10 | 0,06 | -0,01 | -1,01 | dw | 7,41 |  |
| hsa-miR-29b | 11040 | 0,94590 | -0,20 | 0,20 | -0,22 | 0,19 | -0,02 | -1,01 | dw | 12,18 |  |
| hsa-miR-432 | 11111 | 0,94849 | 0,12 | 0,13 | 0,13 | 0,20 | 0,02 | 1,01 | up | 8,25 |  |
| hsa-miR-486-5p | 32946 | 0,95021 | -0,16 | 0,19 | -0,17 | 0,12 | -0,01 | -1,01 | dw | 7,58 |  |
| hsa-miR-369-3p | 29529 | 0,95026 | 0,07 | 0,13 | 0,09 | 0,22 | 0,02 | 1,01 | up | 7,43 |  |
| hsa-miR-671-5p | 42726 | 0,95187 | -0,08 | 0,12 | -0,09 | 0,11 | -0,01 | -1,01 | dw | 7,64 |  |
| hsa-miR-873 | 31436 | 0,95231 | -0,10 | 0,05 | -0,09 | 0,12 | 0,01 | 1,01 | up | 7,57 |  |
| miRPlus_42487 | 42487 | 0,95426 | 0,12 | 0,19 | 0,13 | 0,20 | 0,02 | 1,01 | up | 15,02 |  |
| hsa-miR-630 | 17327 | 0,95836 | -0,05 | 0,15 | -0,04 | 0,21 | 0,01 | 1,01 | up | 8,33 |  |
| hsa-miR-125a-5p | 10928 | 0,95938 | -0,08 | 0,09 | -0,07 | 0,13 | 0,01 | 1,01 | up | 12,64 |  |
| hsa-miR-34b | 42724 | 0,96003 | -0,04 | 0,05 | -0,05 | 0,04 | 0,00 | -1,00 | dw | 10,70 |  |
| hsa-miR-181d | 42840 | 0,96020 | 0,08 | 0,09 | 0,09 | 0,12 | 0,01 | 1,01 | up | 6,77 |  |
| hsa-miR-766 | 21757 | 0,96343 | 0,01 | 0,12 | 0,02 | 0,22 | 0,01 | 1,01 | up | 8,45 |  |
| hsa-miR-30d | 19596 | 0,96382 | 0,19 | 0,16 | 0,18 | 0,14 | -0,01 | -1,01 | dw | 10,40 |  |
| hsa-miR-107 | 10923 | 0,96730 | 0,01 | 0,09 | 0,00 | 0,21 | -0,01 | -1,01 | dw | 10,76 |  |
| hsa-miR-129-5p | 42467 | 0,97113 | 0,18 | 0,12 | 0,18 | 0,10 | 0,01 | 1,00 | up | 10,43 |  |
| hsa-miR-654-3p | 21498 | 0,97128 | 0,22 | 0,08 | 0,22 | 0,15 | -0,01 | -1,00 | dw | 7,68 |  |
| hsa-miR-668 | 42727 | 0,97150 | -0,06 | 0,11 | -0,07 | 0,16 | -0,01 | -1,01 | dw | 7,50 |  |
| miRPlus_17869 | 17869 | 0,97179 | -0,13 | 0,05 | -0,14 | 0,15 | -0,01 | -1,00 | dw | 10,32 |  |
| hsa-miR-634 | 42591 | 0,97305 | -0,15 | 0,10 | -0,15 | 0,08 | 0,00 | 1,00 | up | 10,79 |  |
| hsa-miR-361-5p | 14301 | 0,97747 | 0,13 | 0,10 | 0,13 | 0,10 | 0,00 | 1,00 | up | 10,37 |  |
| hsa-miR-26a | 11030 | 0,97758 | 0,04 | 0,08 | 0,05 | 0,05 | 0,00 | 1,00 | up | 13,74 |  |
| hsa-let-7c | 19004 | 0,98225 | 0,04 | 0,09 | 0,05 | 0,03 | 0,00 | 1,00 | up | 13,70 |  |
| hsa-let-7d | 17750 | 0,98270 | 0,11 | 0,15 | 0,12 | 0,12 | 0,00 | 1,00 | up | 9,52 |  |
| miRPlus_28431 | 28431 | 0,98408 | -0,04 | 0,31 | -0,05 | 0,19 | -0,01 | -1,01 | dw | 12,43 |  |
|  |  |  |  |  |  |  |  |  |  |  |  |
| Annotation | Column ID | T-test | Mean log | Standard error | Mean log ratio | Standard error | Mean Log ratio | Foldchange | Variation | A Mean |  |
|  | Probe Id |  | Control |  | Suicide |  |  | Suicide vs Contrôle |  |  |  |
| miRPlus_17952 | 17952 | 0,98875 | -0,43 | 0,15 | -0,43 | 0,32 | -0,01 | -1,00 | dw | 12,04 |  |
| hsa-miR-921 | 42785 | 0,98904 | -0,19 | 0,22 | -0,19 | 0,32 | 0,01 | 1,00 | up | 12,07 |  |
| hsa-miR-30a | 11048 | 0,98977 | -0,09 | 0,11 | -0,10 | 0,10 | 0,00 | -1,00 | dw | 12,51 |  |
| hsa-miR-144 | 29802 | 0,98982 | -0,06 | 0,20 | -0,05 | 0,19 | 0,00 | 1,00 | up | 8,75 |  |
| hsa-miR-145 | 42641 | 0,99613 | 0,09 | 0,20 | 0,09 | 0,21 | 0,00 | 1,00 | up | 8,60 |  |
| hsa-miR-410 | 11102 | 0,99661 | -0,20 | 0,11 | -0,20 | 0,18 | 0,00 | -1,00 | dw | 8,38 |  |
| hsa-miR-32* | 29575 | 0,99710 | 0,05 | 0,22 | 0,05 | 0,31 | 0,00 | 1,00 | up | 12,06 |  |
| hsa-miR-92b | 17718 | 0,99721 | 0,16 | 0,16 | 0,16 | 0,17 | 0,00 | -1,00 | dw | 8,16 |  |
| hsa-miR-127-3p | 42829 | 0,99945 | 0,07 | 0,17 | 0,07 | 0,24 | 0,00 | -1,00 | dw | 8,52 |  |
| *: microRNA significantly diffentially expressed after correction for multiple testing; FDR 0.05 (Benjamini and Hochberg 1995) | | | | | | | | |  |  |  |
