# Supplementary material for: Regulation of a Truncated Form of Tropomyosin-Related Kinase B (TrkB) by Hsa-miR-185* in Frontal Cortex of Suicide Completers
Source: PLoS One. 2012 Jun 25;7(6):e39301. doi: 10.1371/journal.pone.0039301 (PMC3382618; doi:10.1371/journal.pone.0039301)
Supplement: Table S4 — Mean age, pH and PMI ± standard deviation for the independent larger sample of 55 individuals (17 controls and 38 suicide completers) investigated in the replication study. (DOC) [file pone.0039301.s009.doc]

Supporting Table S4

|  |  | Age | pH | PMI |
| --- | --- | --- | --- | --- |
| Control | Mean | 48,24 | 6,43 | 37,00 |
| n=17 | Stand. Dev. | 22,87 | 0,24 | 19,09 |
| Suicide | Mean | 48,11 | 6,70 | 21,80 |
| n=38 | Stand. Dev. | 15,36 | 0,29 | 23,28 |
