# Supplementary material for: Regulation of a Truncated Form of Tropomyosin-Related Kinase B (TrkB) by Hsa-miR-185* in Frontal Cortex of Suicide Completers
Source: PLoS One. 2012 Jun 25;7(6):e39301. doi: 10.1371/journal.pone.0039301 (PMC3382618; doi:10.1371/journal.pone.0039301)
Supplement: Table S5 — Statistics describing tests for allelic assciations between SNPs located in the fragment containing site 727 from TrkB-T1 3′UTR sequence, and suicide. (DOC) [file pone.0039301.s010.doc]

Supporting Table S5:.

| Name | Assoc Allele | Case,Control Ratio Counts | Case,Control Frequencies | Chi square | Corrected P value |
| --- | --- | --- | --- | --- | --- |
| Novel variant | G | 1:75, 0:34 | 0.013, 0.000 | 0.451 | 1.000 |
| rs7020204 | T | 7:69, 2:32 | 0.092, 0.059 | 0.346 | 1.000 |
| rs45623334 | A | 75:1, 31:3 | 0.987, 0.912 | 3.779 | 0.1557 |
