# Supplementary material for: Regulation of a Truncated Form of Tropomyosin-Related Kinase B (TrkB) by Hsa-miR-185* in Frontal Cortex of Suicide Completers
Source: PLoS One. 2012 Jun 25;7(6):e39301. doi: 10.1371/journal.pone.0039301 (PMC3382618; doi:10.1371/journal.pone.0039301)
Supplement: Supporting Information S1 — Supporting Methods, Supporting Results, Supporting References. (DOC) [file pone.0039301.s011.doc]

**Supporting Methods**

PCR primers used for cloning of *TrkB-T1* 3’UTR sequence are shown in the Supporting Table S1

A first PCR was performed on Genomic cDNA with the primer sets shown in table S1A

A second PCR using primer sets with restriction sites was done on the first purified PCR product with primer sets shown in table S1B.

Restriction sites are underlined. All fragments were digested by SpeI and Hind III enzymes except for fragment containing site 1204 which required SacI and MluI enzymes before cloning in PMIR-report vector..

Primers used to quantify *TrkB* transcripts by real time PCR

The following primers and probe sets commercialized by Applied Biosystems were used:

TrkB-T1 : Hs01093110_m1 ;TrkB-FL : Hs01093094_m1

For TrkB-T2 quantification the following oligonucleotides designed by IDT were used :

primer up, 5’-GGC AGG AAGTGC CCT GGT A-3’

primer dw, 5’-TGG CTA ATG GGC ACT ACATCT G-3’;

Probe:5’-FAM-ATT CTC TCT GTA AGCTCC CCATGT GGC C-3’-TAMRA

Beta-Actin Taqman assay (4310881E) and 18S (4333760F) taqman assay (applied biosystem) were used for normalization.

Sequencing:

*1-Hsa-miR-185* region*

Sequencing of the Hsa-miR-185 region was done using Sanger sequencening at the McGill University Genome Innovation Centre.

We used the following primers:

Forward1 ATCACAGCTGCAGAGCACAC; Forward2: GCGTGGCTTCCTGCAGATGT; Forward3: CAAGAGATGCCCTGAGGAG; Forward4: CTTGGCTTAGGGAGCACACA

Reverse1: agcactcaccatgtggccag

We used the following SNPs assays (Applied Biosystems, CA, USA) to genotype tSNPs in our cohort of 55patients: C__16247305_10 for rs2078749 and C___7543681_20 for rs2008591

2-*TrkB-T1* 3’UTR sequence:

PCR fragment were generated using primers described in table S1.

Genotypes were obtained by Sanger sequencing.

Polymorphisms in Hsa-miR-185 and TrkB-T1 regions were found using Polyphred software [1] available at the McGill University Genome Innovation Centre.

The association study was performed using Haploview 4.2 (Cambridge, MA, USA)

**Supporting Results**

I. Analysis of the Hsa-miR-185 region by sequencing

The sequence encoding Hsa-miR-185 is located on chromosome 22q11.2. To determine if sequence variants could explain the increased Hsa-miR-185* in frontal cortex from suicide completers, we sequenced the DNA encoding Hsa-miR-185 microRNA by proceeding in two steps.

First, in a subgroup of 10 suicides with low TrkB-T1 expression and 5 controls we sequenced a 1682 bp fragment (comprising the 82 bp sequencing coding for the Hsa-mir-185, 1084 upstream bases and 516 in the downstream region) in order to detect possible sequence variants. We found 7 SNPs, four of them already annotated in Genebank (rs887205; rs9605051; rs2078749 and rs2008591). Of these, rs2078749 and rs2008591 meet criteria for tag SNPs. The three other SNPs not annotated by Genbank were not informative (Figure S3A).

In a second step, we genotyped the two tSNPs, which capture genetic information in this region, in the 55 patients included in this study. We found no evidence of significant association (either allelic, genotypic or haplotypic) (Figure S3B and S3C) between any of these two tSNPs and suicide. Furthermore, we found no significant differences between genotypes and Hsa-miR-185* expression levels Figure S3D and Figure S3E).

These data suggest that the upregulation of the Hsa-miR-185* in suicide completers is unlikely to be a result of genetic variants in the sequence coding for this microRNA.

II. Analyses of polymorphisms in *TrkB-T1* 3’UTR sequence

Genetic variants may help explain part of our results. For instance, Kholi et al have recently shown that polymorphisms in the TrkB gene are associated with a lifetime history of suicide attempts in depressed patients[2]. To better understand if variants in the *TrkB-T1* 3’UTR sequence may account for at least part of our results, we sequenced the 3’UTR DNA regions where miR-185* is predicted to bind, as well as their adjacent 100bp upstream and 100bp downstream sequences. We found three polymorphisms in our sample. Two of these polymorphisms are known variants annotated by Genebank as rs7020204 and rs45623334. They are located downstream of the putative binding site. The third variant-a novel A/G polymorphism- is located in the sequence in which the microRNA binds. We found no significant difference in the distribution of these variants between suicides and controls (see Supporting Table S5)

III. Analysis of TrkB-FL expression level by Western blot

A TrkB-FL decrease in suicide completers has been described in the literature [3]. To assess whether our *TrkB-T1* results were not a result of an overall TrkB downregulation, we investigated BA10 levels of TrkB-FL, the major TrkB transcript, by Western blot. We found no significant difference in protein levels between groups (t=1.047 df=6 p=0.335), (Figure S4).

IV. Expression level of Hsa-miR-185* in cerebellum

Previously, we showed no significant difference in *TrkB-T1* expression in cerebellum [4] (a region known not to be involved in suicide pathophysiology) by comparing control and suicide completers. In order to know whether Hsa-miR-185* deregulation is specific from frontal cortex or not, we quantified this microRNA in the cerebellum of the patients included in the microarray study. We found no significant difference between suicides and controls (t=-2.070 df=6, p=0.11).

V. Expression level of microRNA that could bind *TrkB-T1* 3’ UTR sequence

In our study, we focused on the interaction between the microRNA Hsa-miR-185* and the *TrkB-T1* transcript based on the results of our microarray screening and subsequent *in silico* analyses. However, other microRNAs may modulate *TrkB-T1* expression levels through an interaction with its 3’UTR sequence. Based on the potential interactions as estimated by RNA22, we selected candidate microRNAs that could bind *TrkB-T1*. Candidate microRNAs were chosen according to the following criteria:

1. Brain expression detected in all the samples investigated in the microarray experiment.
2. Upregulation in suicide completers
3. Intensity signal similar to the one presented by Hsa-miR-185* on the array.
4. Theoretical ability to bind the *TrkB-T1* 3’UTR sequence (Folding energy below -25kcal/mol; seed comprising 7 or 8 pb with 2 G:U allowed; these parameters were similar to those used when investigating TrkB-T1 binding potential for Hsa-miR-185*).
5. Candidates with similar annotation to the version 11.0 of mirBase used for the microarray experiment.

Hsa-423-5p and Hsa-193a-5p, respectively at rank 10 and rank 30 from our microarray table, fit all the criteria above. However, no significant change was found in Hsa-miR-423-5p (t=-0.11 df=52 p=0.913) or in Hsa-miR-193a-5p (t=0139 df=52 p=0.89) expression levels between suicides and controls

VI. Analysis of the confounding effect of ante-mortem medication on Hsa-miR-185* expression level

We investigated the potential effect of antidepressant medication on Hsa-miR-185* expression levels. Out 38 suicides completers, 15 of them had a prescription of antidepressant at least once in the three months before death. Toxicological reports revealed the presence of antidepressants at the time of death for 9 of these cases. We found no significant difference in Hsa-miR-185* expression levels between patients with or without a prescription for antidepressant during the last 3 months of life (t=-0.289; df=36; p= 0.774) nor between patients with or without antidepressant detected by toxicology at the time of death (t=-1.093; df=13; p=0.294). These data suggest that the expression level of Hsa-miR-185* does not depend on antidepressant medication.

VII. Suicide and substance use

Use of psychoactive substances is frequently seen in suicide completers.. To assess the potential effect of substance use on our results, we investigated the relationship of alcohol and illicit drugs on Hsa-miR-185* levels. Toxicological analyses performed on our samples indicated that 23.6% of patients had detectable levels of alcohol and 21.8% of cocaine and other illicit drugs. ANCOVA analyses controlling for the effects of alcohol and drugs showed that substance does not account for the differences in Hsa-miR-185* expression between groups, as the effect of substance is not significant (F = 0.16; P = 0.693), while the difference between suicides and controls remains significant (F = 6.49; P = 0.014) when controlling for substance. These data suggest that even if substance use is a risk factor for suicide behaviour, this does not explain the significant difference of Hsa-miR-185* expression level between suicides and controls.

VIII. Confounding effect of acute or chronique Illnesses on the expression level of Hsa-miR-185*.

To investigate the possible effect of illness duration on our results, we compared cases with a chronic and acute illness course. We found no significant difference in Hsa-miR-185* expression level between these two groups (t=-0.571; df=31; p=0.572)

**Supporting References**

1. Nickerson DA, Tobe VO, Taylor SL (1997) PolyPhred: automating the detection and genotyping of single nucleotide substitutions using fluorescence-based resequencing. Nucleic Acids Res 25: 2745-2751.

2. Kohli MA, Salyakina D, Pfennig A, Lucae S, Horstmann S, et al. (2010) Association of genetic variants in the neurotrophic receptor-encoding gene NTRK2 and a lifetime history of suicide attempts in depressed patients. Arch Gen Psychiatry 67: 348-359.

3. Pandey GN, Ren X, Rizavi HS, Conley RR, Roberts RC, et al. (2008) Brain-derived neurotrophic factor and tyrosine kinase B receptor signalling in post-mortem brain of teenage suicide victims. Int J Neuropsychopharmacol 11: 1047-1061.

4. Ernst C, Deleva V, Deng X, Sequeira A, Pomarenski A, et al. (2009) Alternative splicing, methylation state, and expression profile of tropomyosin-related kinase B in the frontal cortex of suicide completers. Arch Gen Psychiatry 66: 22-32.
